# DNA hypomethylation of INHBA promotes tumor progression and predicts prognosis and immune status of gastric cancer

**DOI:** 10.1186/s41065-024-00347-7

**Published:** 2024-11-14

**Authors:** Xueying Li, Haizhong Jiang, Yangbo Fu, Qiying Hu, Xianlei Cai, Guoqiang Xu

**Affiliations:** 1 Department of Gastroenterology, First Affiliated Hospital, Ningbo University, Ningbo, Zhejiang 315000 China; 2 Department of Gastroenterology, First Affiliated Hospital, School of Medicine, Zhejiang University, Hangzhou, 310003 China; 3 Ningbo Key Laboratory of Translational Medicine Research on Gastroenterology and Hepatology, Ningbo, Zhejiang 315000 China; 4 Department of Gastrointestinal Surgery, The Ningbo Medical Center Lihuili Hospital, Ningbo, Zhejiang 315000 China

**Keywords:** INHBA, DNA methylation, Prognosis, Immune status, Gastric cancer

## Abstract

**Objective:**

Gastric cancer (GC) is characterized by its high malignancy and poor prognosis. However, the role of Inhibin subunit beta A (INHBA) in GC remains insufficiently understood. This study aims to comprehensively evaluate the clinical significance, biological roles, and possible mechanisms of INHBA in GC.

**Methods:**

Expression levels and survival analyses of the Inhibin beta family were assessed using online databases. A prediction model based on INHBA was developed. In addition, the associations between INHBA expression and immune status, and chemotherapy sensitivity were explored. In vitro experiments were conducted to investigate the biological impact of INHBA on GC cells. Pyrosequencing and the DNA methylation inhibitor, 5-AZA-2’-deoxycytidine (5-AZA-dC) were employed to elucidate the mechanisms underlying INHBA function.

**Results:**

Our findings revealed that INHBA exhibited high expression in GC patients, and elevated INHBA expression correlated with worse outcomes. We developed a novel nomogram incorporating INHBA, age, and tumor node metastasis (TNM) stage to predict the prognosis of GC patients. Additionally, INHBA was found to be associated with suppressed infiltration of immune cells and chemosensitivity. Functionally, INHBA promoted the proliferation and invasiveness of GC cells. Mechanistically, pyrosequencing revealed DNA Hypomethylation of INHBA in the first exon region, and the effects of INHBA silencing were rescued by 5-AZA-dC treatment.

**Conclusion:**

Our study suggests that DNA hypomethylation of INHBA contributes to the progression of GC. Furthermore, INHBA holds promise as a valuable biomarker for prognostic evaluation and immune status prediction in GC patients.

**Supplementary Information:**

The online version contains supplementary material available at 10.1186/s41065-024-00347-7.

## Introduction

Gastric cancer (GC) holds a significant position, ranking sixth among the most common cancers globally and serving as the third leading contributor to cancer-related mortality worldwide [1]. GC is characterized by high malignancy and poor prognosis [2]. Unfortunately, chemotherapy’s effectiveness against advanced GC remains unsatisfactory [2], and the development of targeted drugs for gastric cancer has been sluggish [3]. Therefore, the exploration of new biomarkers for therapeutic targets holds great promise in addressing this clinical challenge.

The signaling pathway of transforming growth factor-beta (TGF-β) plays a pivotal position in orchestrating diverse cellular functions, encompassing cell viability, differentiation, and immunological modulation [4]. Immunotherapy has emerged as a promising therapeutic option for GC, but its efficacy is hindered by the immunosuppressive microenvironment fostered by TGF-β signaling [4]. Consequently, targeting the TGF-β signaling pathway holds significant potential for enhancing the effectiveness of immunotherapy in GC.

The Inhibin beta family comprises genes encoding members of the TGF-β superfamily of proteins and has been implicated in cancer progression. This family includes four subtypes: Inhibin subunit beta A (INHBA), Inhibin subunit beta B (INHBB), Inhibin subunit beta C (INHBC), and Inhibin subunit beta E (INHBE). Among them, INHBA has garnered significant attention. Chen et al. [5] discovered that the silencing of INHBA exhibited a suppressive effect on GC progression by inactivating the TGF-β signaling pathway. Qiu et al. [6] showed that CircTHBS1 promotes GC progression through the augmentation of INHBA mRNA expression as well as its stability. Kumar et al. [7] constructed a single-cell atlas in GC and identified cancer-associated fibroblast subpopulations marked by high INHBA expression.

Epigenetic modifications, particularly DNA methylation, have been recognized to play a pivotal role in the development and progression of GC [8]. DNA methylation is a reversible DNA modification involving the addition of a methyl group to cytosine residues within CpG dinucleotides, leading to gene silencing [9]. Abnormal DNA methylation of oncogenes can result in their overexpression, contributing to gastric carcinogenesis. However, the regulatory characteristics of DNA methylation within the INHBA in the context of GC remain poorly understood.

This study aims to elucidate the clinical relevance, biological roles, and DNA methylation modification of INHBA in GC, with a specific emphasis on exploring its potential as a novel biomarker and therapeutic target in the context of GC.

## Materials and methods

### Cells, specimens, and patients

Two human gastric cancer cell lines, HGC-27 (CSTR:19375.09.3101HUMTCHu22) and AGS (CSTR: 19375.09.3101HUMTCHu232) were obtained from the Shanghai Institutes of Biological Sciences (Shanghai, China). Authentication of cell lines was conducted using Short Tandem Repeat (STR) testing.

A total of ten pairs of specimens were collected from GC patients who underwent surgery in 2022 at Ningbo First Hospital for pyrosequencing analysis. The study was approved by the Institutional Ethics Committee in Ningbo First Hospital, and written informed consent was obtained from all patients in accordance with the principles outlined in the Declaration of Helsinki.

RNA-sequencing expression profiles and relevant clinical information were sourced from The Cancer Genome Atlas (TCGA) repository (https://portal.gdc.cancer.gov). The TCGA data were acquired and preprocessed utilizing the online bioinformatics analysis platform, Assistant for Clinical Bioinformatics (ACBI), which is accessible at https://www.aclbi.com/. Ultimately, a cohort of 375 patients diagnosed with stomach adenocarcinoma (STAD) was collected and analyzed with the assistance of ACBI. The dataset included age, gender, tumor node metastasis (TNM) stage, vital status, and follow-up time (in years) as clinical parameters. To normalize the RNA count data, transcripts per million (TPM) values were calculated. The STAD patients were categorized into two groups based on their RNA expression levels, namely high and low expression groups, using the median RNA expression as the threshold value. After removing cases with missing data, a total of 354 patients were included in the univariate and multivariate Cox regression analysis.

### Bioinformatics analysis

We utilized various online bioinformatics analysis tools for comprehensive data analysis. The ACBI platform was employed for expression difference and correlation analysis based on TCGA and The Genotype-Tissue Expression (GTEx) databases. The Search Tool for the Retrieval of Interacting Genes/proteins (STRING) (https://cn.string-db.org/) was utilized for protein-protein interaction analysis. Within the same gene family, members often exhibit a certain degree of functional similarity. Utilizing STRING, the interconnections and complementary roles among the inhibin beta subunit family were illustrated. By further focusing on a specific gene, we could gain an understanding of the shared characteristics of this gene family. Kaplan-Meier Plotter (http://kmplot.com/) served as the platform for conducting Kaplan-Meier analysis, integrating various public datasets [10]. For the analysis of DNA methylation levels based on TCGA data, we utilized The University of Alabama at Birmingham CANcer data analysis Portal (UALCAN) [11, 12] (https://ualcan.path.uab.edu), the cBio Cancer Genomics Portal (cBioPortal) [13, 14] (http://www.cbioportal.org/), and the University of California Santa Cruz Xena (UCSC Xena) [15] (http://xena.ucsc.edu/). TISIDB [16] (http://cis.hku.hk/TISIDB/) and the Tumor IMmune Estimation Resource (TIMER) [17] (https://cistrome.shinyapps.io/timer/) were employed for analyzing immune status based on TCGA data. Finally, the Genomics of Drug Sensitivity in Cancer (GDSC) [18, 19] platform was utilized for predicting drug sensitivity. The GSE62254 dataset [20], comprising microarray profiles from 300 gastric tumors, as reported by the Asian Cancer Research Group, was utilized for bioinformatics analysis to validate the associations between INHBA and immune infiltration. CIBERSORT was used to calculate the abundance of immune cells in each sample. Corr.test function from R studio was used to calculate correlation coefficient between gene expression and immune cells, and R package ‘ggpubr’ was used for visualization.

### Development of the risk-prediction model

To evaluate the clinical significance of INHBA, we developed a nomogram, termed the INHBA model, which integrated INHBA expression and relevant clinical characteristics. This model was constructed by using multivariate Cox regression analysis to predict the overall survival (OS) of individual patients with STAD at both 3 and 5 years. To provide a comprehensive analysis, we also developed a separate model according to the pathologic TNM stage, referred to as the TNM model. The predictive performance of these models was assessed using time-dependent receiver operating characteristic (ROC) curves, which capture the evolving predictive capacity over time [21, 22]. Furthermore, we conducted a decision curve analysis (DCA) to assess the clinical utility of the INHBA model in comparison with the TNM models. The DCA graph quantified the potential net benefit at various threshold probabilities, enabling a direct comparison between the INHBA model and the TNM models [23].

## DNA methylation-related experiments

### Pyrosequencing testing

Genomic DNA extraction was carried out using a genomic DNA extraction kit (QIAGEN), and bisulphite conversion was performed using the Qiagen EpiTect Bisulfite Kit (QIAGEN. Cat. No.59104). Pyrosequencing testing was conducted according to the manufacturer’s protocol [24]. In brief, the pretreated substrate mixture, enzyme mixture, and four types of dNTPs (QIAGEN) were added to the reagent chamber based on the doses calculated by the Pyrosequencing software, and then the reagent chamber along with the 96-well reaction plate was placed into a Pyrosequencing detector (PyroMark Q96 ID, QIAGEN) for the reaction. The methylation status of each site was analyzed using the Pyro Q-CpG software. Primer design was carried out using PyroMark Assay Design 2.0. Finally, three independent primers were used to assess the DNA methylation level in the first exon region of INHBA. Primer-1 contained 6 sites, Primer-2 contained 11 sites, and Primer-3 contained 9 sites of DNA methylation. All primers are listed in Supplementary Table 1.

### Rescue experiments

To perform the rescue experiment, we employed the DNA methylation inhibitor 5-AZA-2’-deoxycytidine (5-AZA-dC) [25]. The HGC-27 and AGS cell lines were treated with 2 µmol/L 5-AZA-dC for 72 h. Subsequently, we reassessed the expression of INHBA, as well as tumor growth and migration/invasion capabilities, using cell proliferation assays, colony formation assays, wound healing assays, and transwell assays.

### Statistical analysis

For normally distributed quantitative data, the two-tailed Student’s t-test was applied, otherwise, the Mann-Whitney U test was employed. The chi-square test was used for evaluating qualitative data. A Spearman’s correlation analysis was conducted to examine the underlying correlations. For comparing survival disparities, a Kaplan-Meier survival analysis accompanied by the log-rank test was employed. Furthermore, both univariate and multivariable Cox regression analyses were conducted to estimate the hazard ratio (HR) associated with potential risk factors. The procedure for building the model was described in our previous studies [26, 27].

Each experiment was independently repeated three times. The data are presented as means ± standard deviations (SDs). Statistical analyses were conducted using GraphPad Prism 8.0 and R software for Windows (version 4.0.3), with significance thresholds set as follows: *p < 0.05, **p < 0.01, ***p < 0.001, and ****p < 0.0001. Additional methodologies and details are outlined in the Supplementary Methods section, along with Supplementary Tables 2–4.

## Results

### High expression of inhibin beta family genes is associated with poor outcomes in STAD

**Fig. 1:**
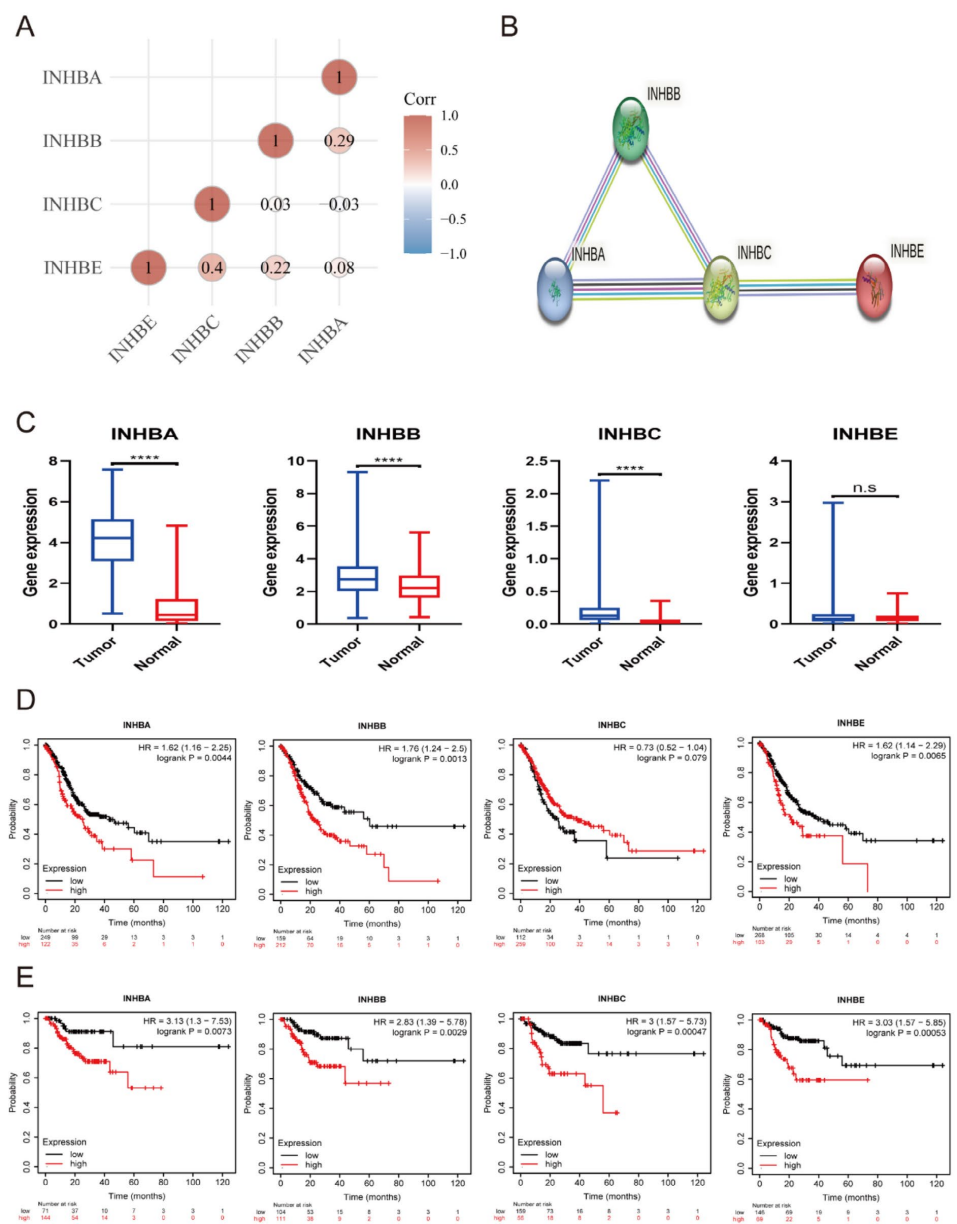
Bioinformatics analyses of inhibin beta subunit family in STAD. **(A)** The correlations between the genes of inhibin beta subunit family; **(B)** The protein-protein associations between inhibin beta subunit family genes; **(C)** The expression of inhibin beta subunit family genes mRNA was compared between tumors and normal tissues based on TCGA and GTEx datasets (**** *p*<0.0001; Mann-Whitney test); **(D)** Kaplan-Meier analysis of overall survival for the inhibin beta subunit family genes (log-rank test); **(E)** Kaplan-Meier analysis of recurrence-free survival for the inhibin beta subunit family genes (log-rank test)

To evaluate the clinical significance of inhibin beta family genes (INHBA, INHBB, INHBC, and INHBE) in STAD, we investigated their interrelations (Fig. 1A and B) and compared their expression levels between tumor and normal tissues (Fig. 1C). We observed significant upregulation of INHBA, INHBB, and INHBC in STAD. Kaplan-Meier survival analysis revealed that high expression of inhibin beta family genes was associated with poor OS (Fig. 1D) and recurrence-free survival (RFS) (Fig. 1E) in STAD. Given the functional similarities among the proteins encoded by these genes and the pronounced difference in INHBA expression between tumor and normal tissues, we focused our attention on INHBA.

### INHBA-associated nomogram enhances the prediction accuracy of the TNM system

**Fig. 2:**
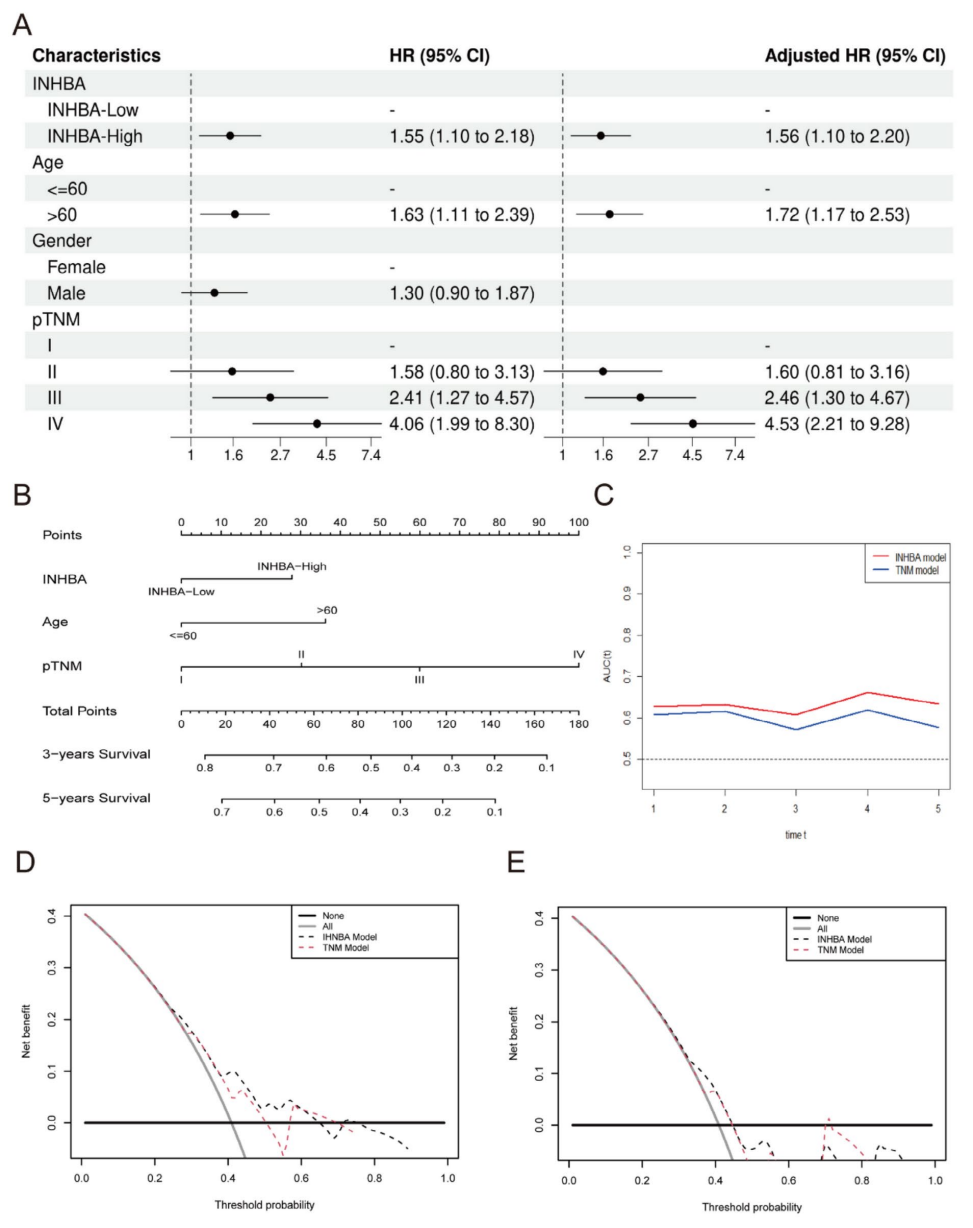
INHBA-associated nomogram enhances the prediction accuracy of the TNM system. **(A)** The forest plot of univariate and multivariate Cox regression analysis for INHBA; **(B)** A Nomogram to predict 3-years and 5-years overall survival (OS) based on INHBA expression and Clinicopathologic features. Each risk factor corresponded to a point by drawing a line straight upward to the points axis. The sum of the points located on the total points axis indicated the probability of OS by drawing a line straight down to the survival axis; **(C)** The time dependent ROC curves comparing the INHBA model with the TNM model; **(D)** The decision curve analysis (DCA) curves depict the clinical value of the INHBA models to predict 3-years OS when compared with the TNM model; **(E)** The DCA curves depict the clinical value of the INHBA models to predict 5-years OS when compared with the TNM model

Based on TCGA data, we integrated clinical parameters and INHBA expression, finding that patients with high INHBA expression had a higher proportion of pathological T4 stages (Table 1). Univariate and multivariate Cox regression analyses demonstrated that INHBA was a significant independent risk factor [HR = 1.56; 95% confidence interval (CI): 1.10–2.20], along with TNM stage and age (Fig. 2A; Table 2).

**Table 1:** Baseline characteristics

Characteristics	levels	INHBA-Low (*N* = 172)	INHBA-High (*N* = 172)	*p*
Age	<=60	60 (34.9%)	55 (32%)	0.648
	> 60	112 (65.1%)	117 (68%)	
Gender	Female	65 (37.8%)	58 (33.7%)	0.500
	Male	107 (62.2%)	114 (66.3%)	
Pathological T	T1	16 (9.3%)	2 (1.2%)	0.004
	T2	38 (22.1%)	34 (19.8%)	
	T3	79 (45.9%)	83 (48.3%)	
	T4	39 (22.7%)	53 (30.8%)	
Pathological N	N0	51 (29.7%)	55 (32%)	0.969
	N1	48 (27.9%)	46 (26.7%)	
	N2	36 (20.9%)	36 (20.9%)	
	N3	37 (21.5%)	35 (20.3%)	
Pathological M	M0	152 (88.4%)	155 (90.1%)	0.728
	M1	20 (11.6%)	17 (9.9%)	
Status	Alive	113 (65.7%)	94 (54.7%)	0.047
	Dead	59 (34.3%)	78 (45.3%)	

**Table 2:** Univariate and multivariate analysis of influencing factors (Cox regression)

	Univariable					Multivariable				
Characteristic	N	Event N	HR	95% CI	p-value	N	Event N	HR	95% CI	p-value
INHBA										
INHBA-Low	172	59	1.0	—		172	59	1.0	—	
INHBA-High	172	78	1.55	1.10, 2.18	0.012	172	78	1.56	1.10, 2.20	0.012
Age										
<=60	115	36	1.0	—		115	36	1.0	—	
> 60	229	101	1.63	1.11, 2.39	0.012	229	101	1.72	1.17, 2.53	0.005
Gender										
Female	123	42	1.0	—						
Male	221	95	1.30	0.90, 1.87	0.16					
pTNM										
I	49	11	1.0	—		49	11	1.0	—	
II	112	34	1.58	0.80, 3.13	0.19	112	34	1.60	0.81, 3.16	0.18
III	146	68	2.41	1.27, 4.57	0.007	146	68	2.46	1.30, 4.67	0.006
IV	37	24	4.06	1.99, 8.30	< 0.001	37	24	4.53	2.21, 9.28	< 0.001

Driven by these findings, we formulated a prognostic model termed the “INHBA model,” which incorporates INHBA expression levels, age, and TNM staging. Additionally, we developed a visually intuitive nomogram (Fig. 2B) to facilitate comprehension. To evaluate the clinical utility of INHBA, we established a novel model that directly compares with the conventional TNM staging system (the “TNM model”). Our analysis revealed that the INHBA model, assessed through the time-dependent ROC curve, exhibited superior predictive accuracy compared to the TNM model (Fig. 2C). However, this method could not provide comprehensive statistical metrics, and we need to interpret the results with caution.

Furthermore, the clinical value of the INHBA model was compared with the TNM model using DCA (decision curve analysis) curves. The INHBA model consistently demonstrated a superior net benefit for 3-year and 5-year OS (Fig. 2D and E). These findings highlight the novel biomarker potential of INHBA for predicting outcomes in STAD and its promising translational value in clinical settings.

### Association between INHBA, immunotherapy, and immune cell infiltration

**Fig. 3:**
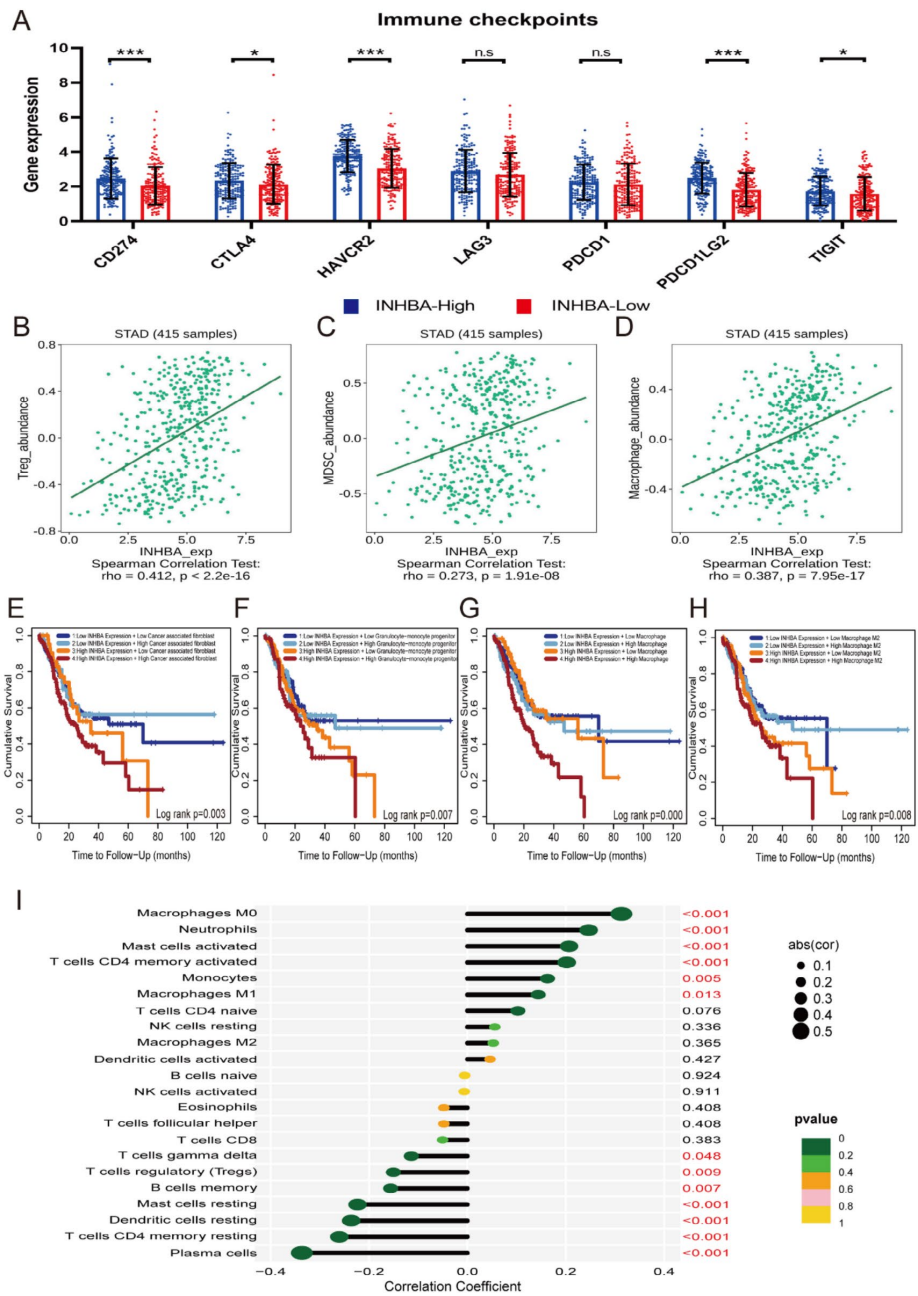
The effect of INHBA on predicting immune status of STAD patients. **A.** The correlations between INHBA expression and immune checkpoints expression (* *p*<0.05, *** *p*<0.001; Mann-Whitney test); **B-D.** Bioinformatics analyses showed a positive correlation between INHBA expression and the abundance of immunosuppressive T cells (Spearman correlation test): (**B**) Treg; (**C**) MDSC; (**D**) Macrophage; **E-H.** Kaplan-Meier analyses of overall survival based on INHBA expression and immune cell infiltration. The results showed that patients with high INHBA expression and high cancer associated fibroblast (**E**), granulocyte-monocyte progenitor (**F**), macrophage (**G**), and M2 macrophage (**H**) infiltration had the worst prognosis. **I**. The correlations between INHBA and immune infiltration were validated using GSE62254

To explore the role of INHBA in immunotherapy, we examined the correlation between its expression and common immune checkpoint-related genes, including CD274 (Programmed cell death 1 ligand 1, PD-L1), Hepatitis A virus cellular receptor 2 (HAVCR2), Cytotoxic T-lymphocyte-associated protein 4 (CTLA4), Programmed cell death 1 ligand 2 (PDCD1LG2), Lymphocyte-activation gene 3 (LAG3), Programmed cell death 1 (PDCD1), and T cell immunoreceptor with Ig and ITIM domains (TIGIT). The expression levels of CD274, CTLA 4, HAVCR2, PDCD1LG2, and TIGIT was significantly higher in patients in the INHBA-high group (Fig. 3A).

Furthermore, bioinformatics analyses revealed a positive association between INHBA expression and the abundance of regulatory T cells (Tregs), myeloid-derived suppressor cells (MDSCs), and Macrophages (Fig. 3B-D), which typically play an immunosuppressive role in the tumor microenvironment. Moreover, Kaplan-Meier analyses of OS based on INHBA expression and immune cell infiltration demonstrated that high INHBA expression, along with infiltration of cancer-associated fibroblasts (Fig. 3E), granulocyte-monocyte progenitors (Fig. 3F), macrophages (Fig. 3G), and M2 macrophages (Fig. 3H) were associated with a worse prognosis. In addition, we utilized GSE62254 to validate the associations between INHBA and immune infiltration. We found that the expression of INHBA was correlated with multiple types of immune cells (Fig. 3I). These findings indicate that INHBA is linked to the immune status of GC patients and may serve as a potential biomarker for predicting the efficacy of immunotherapy.

### The association between INHBA and chemosensitivity

**Fig. 4:**
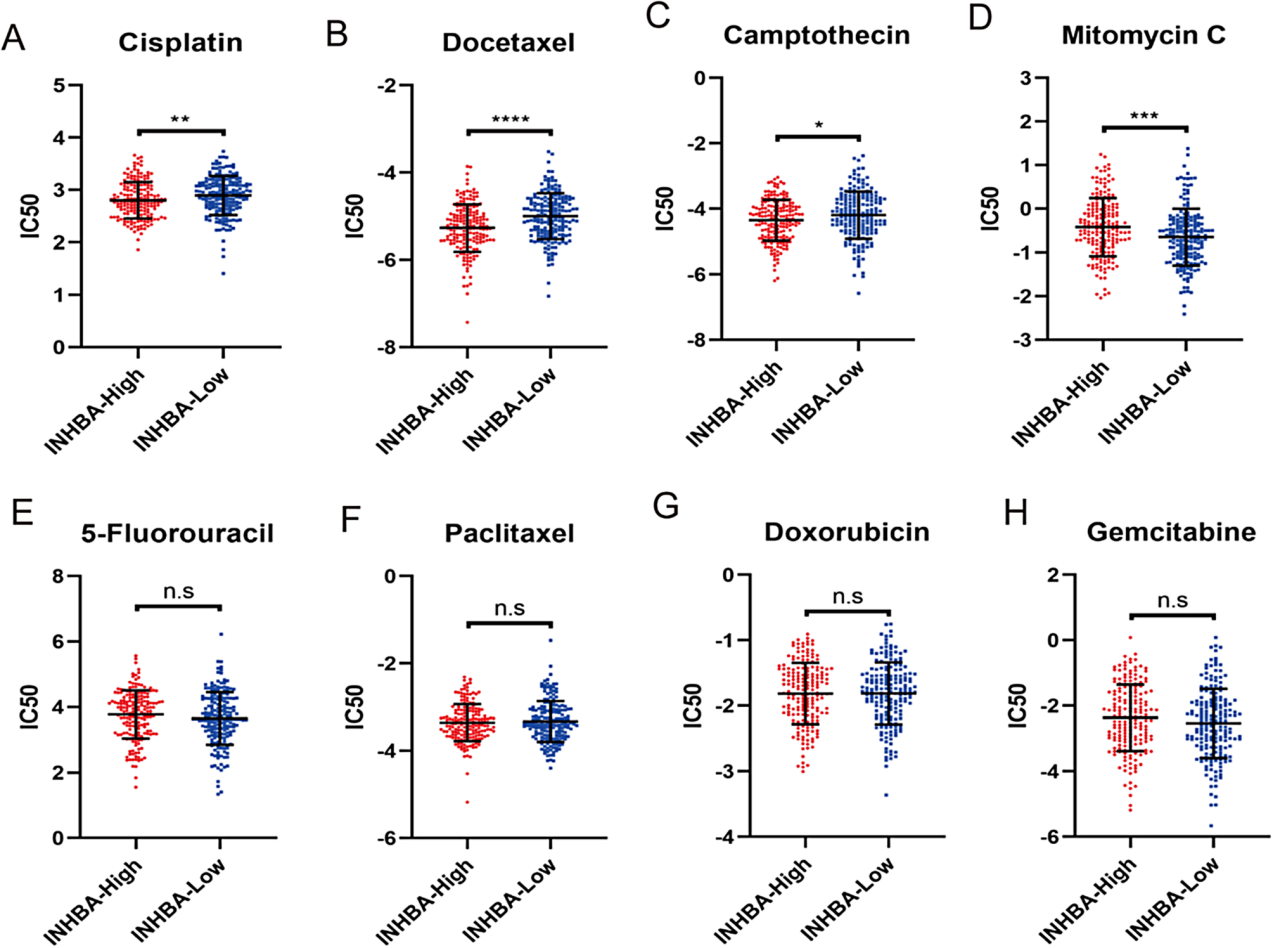
The effect of INHBA on predicting chemosensitivity. **A-H**. The IC50 values of eight commonly used chemotherapy drugs in clinical practice were compared between the INHBA-high and INHBA-low groups (* *p*<0.05, ** *p*<0.01, *** *p*<0.001, **** *p*<0.0001; Mann-Whitney test). **(A)** Cisplatin; **(B)** Docetaxel; **(C)** Camptothecin; **(D)** Mitomycin C; **(E)** 5-Fluorouracil; **(F)** Paclitaxel; **(G)** Doxorubicin; **(H)** Gemcitabine

Chemotherapy is the primary treatment approach for patients with advanced GC. To further investigate the impact of INHBA expression on chemosensitivity, we assessed the effect of INHBA expression on drug sensitivity using the Genomics of Drug Sensitivity in Cancer (GDSC) database. Our analysis revealed that patients in the INHBA-high group had lower half-maximal inhibitory concentration (IC50) values for cisplatin, docetaxel, and camptothecin, indicating greater sensitivity to these drugs. Conversely, the INHBA-high group displayed higher IC50 values for mitomycin C (Fig. 4A-D). No significant differences were observed between the INHBA-high and the INHBA-low groups for 5-fluorouracil (5-Fu), paclitaxel, doxorubicin, and gemcitabine (Fig. 4E-H). These findings suggest that patients with high INHBA expression may benefit from cisplatin, docetaxel, and camptothecin as initial treatment options, as they are more likely to exhibit favorable sensitivity to these drugs.

### INHBA promotes growth and migration/invasion capability of gastric cancer cells in vitro

**Fig. 5:**
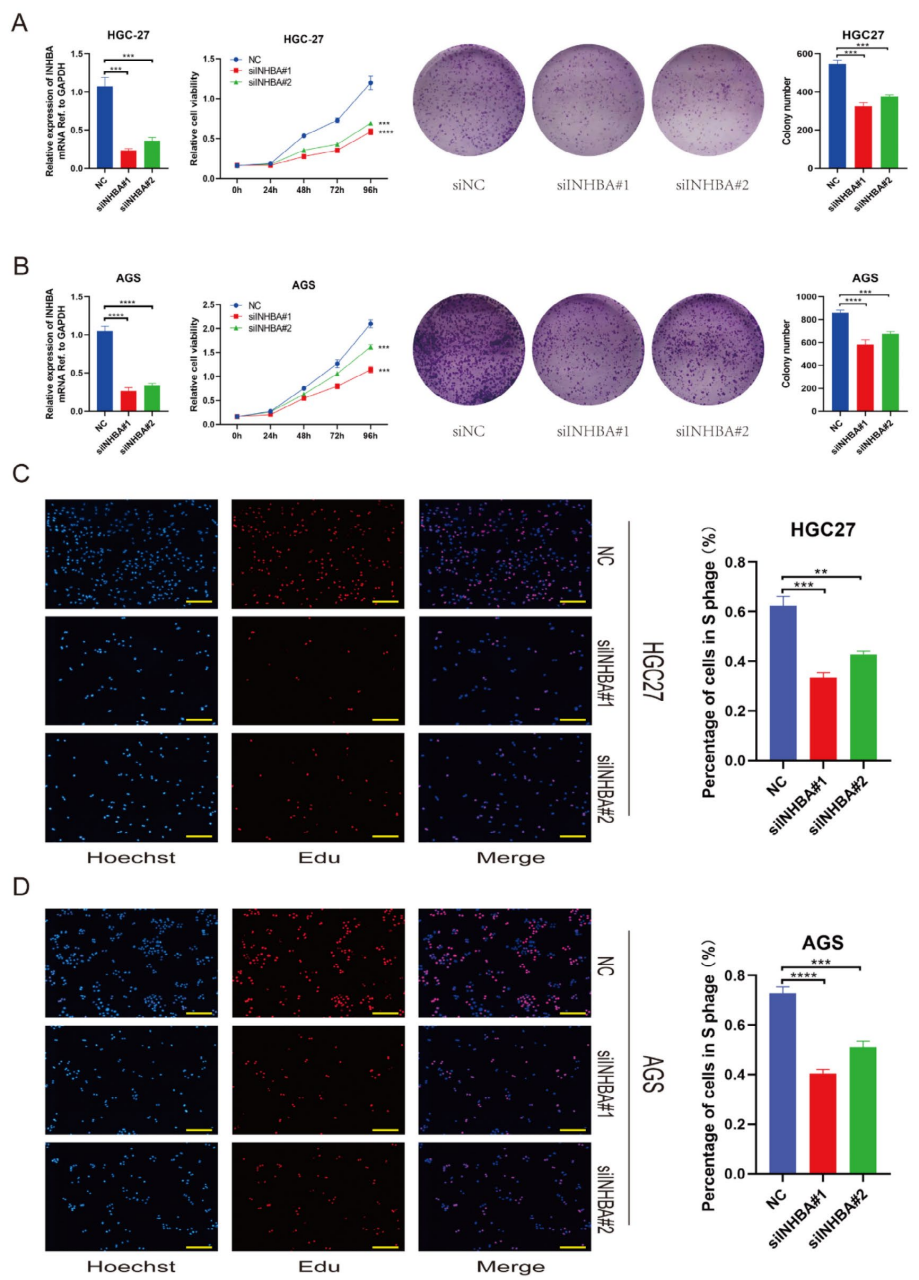
INHBA promotes tumor growth capability of gastric cancer cells in vitro. **A-B.** Negative control or siRNA (si-INHBA#1 and #2) was transfected into HGC27 (**A**) and AGS (**B**), respectively. The efficiency of knockdown was tested by RT-qPCR and the proliferation capacities of STAD cells were detected by CCK-8 and colony formation assays (*** *p*<0.001, **** *p*<0.0001; *t*-test); **C-D.** Edu assay was applied to compare the proliferation abilities of HGC27 (**C**) and AGS cells (**D**) (scale bar, 200 μm); bar charts showed the percentage of cells in S phage based on the results of Edu assay (** *p*<0.01, *** *p*<0.001, **** *p*<0.0001; *t*-test). The data are presented as means ± SD

To elucidate the biological functions of INHBA in gastric cancer, we employed the HGC-27 and AGS cell lines to establish INHBA-knockdown models. The relative mRNA expression levels of INHBA in these two cell lines are shown in Supplementary Fig. 1. The transfection efficiency was validated (Fig. 5A, B and Supplementary Fig. 2A). Our results revealed that the knockdown of INHBA inhibited the proliferation capability of GC cells, as demonstrated by CCK-8 and colony formation assays (Fig. 5A and B). Furthermore, the Edu assay confirmed that the knockdown of INHBA inhibited gastric cancer cell growth in vitro (Fig. 5C and D).

**Fig. 6:**
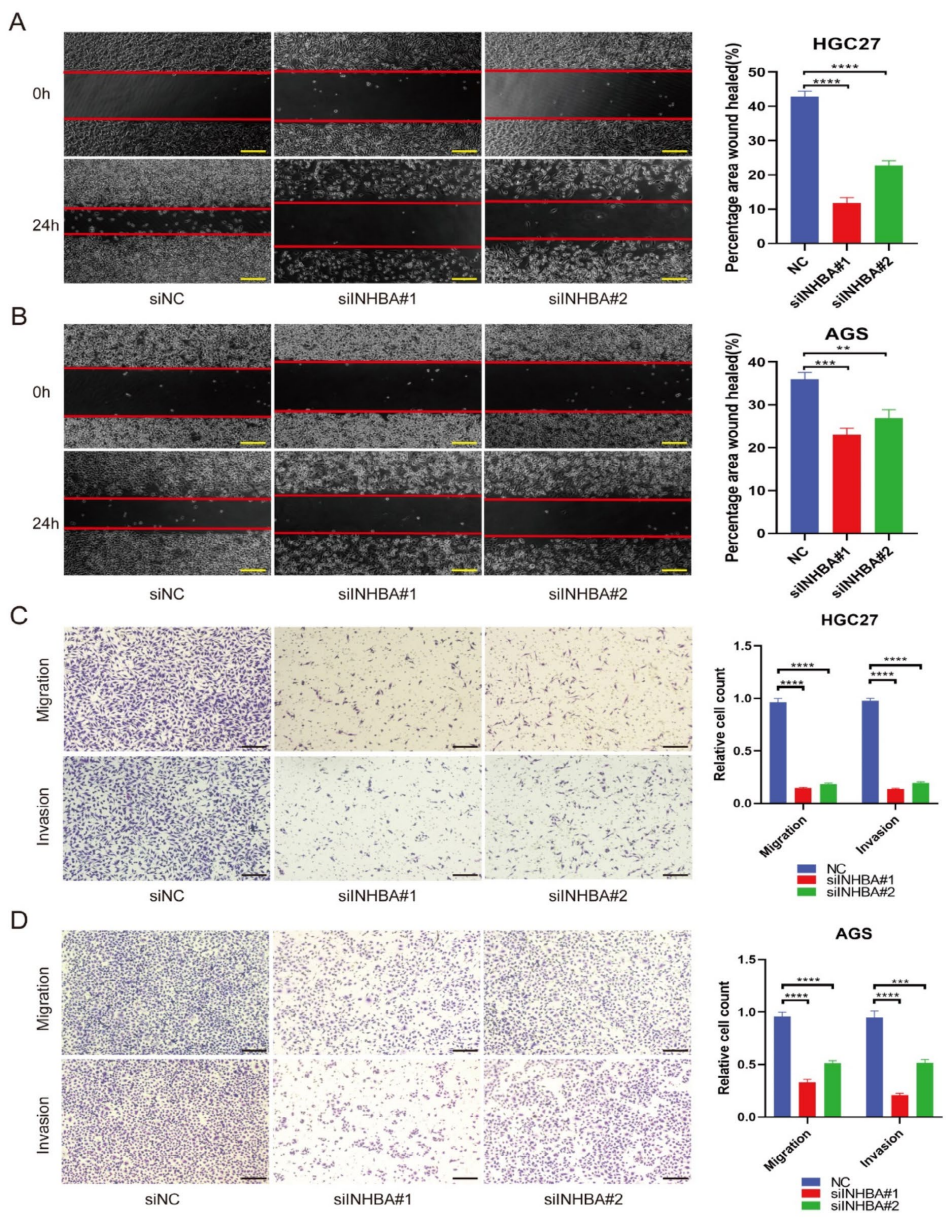
INHBA promotes tumor migration / invasion capability of gastric cancer cells in vitro. **A-B.** Wound healing assays were performed to compare the migration capabilities of HGC27 (**A**) and AGS cells (**B**) (scale bars, 200 μm); the percentage of healed area were quantified by bar charts (** *p*<0.01, *** *p*<0.001, **** *p*<0.0001; *t*-test); **C-D.** Transwell assays were applied to detect the migration and invasion abilities of HGC27 (**C**) and AGS cells (**D**) after silencing INHBA (scale bars, 200 μm); bar charts showed the relative count of two groups of STAD cells which passed through the chamber membranes, compared to negative control groups (*** *p*<0.001, **** *p*<0.0001; *t*-test); The data are presented as means ± SD

To explore the impact of INHBA on migration and invasion, we performed wound-healing assays and observed that INHBA promoted the migration of gastric cancer cells (Fig. 6A and B). Transwell assays demonstrated that INHBA deficiency hindered the migration and invasion abilities of GC cells (Fig. 6C and D).

### DNA hypomethylation enhances INHBA expression and activates the TGF-β pathway

**Fig. 7:**
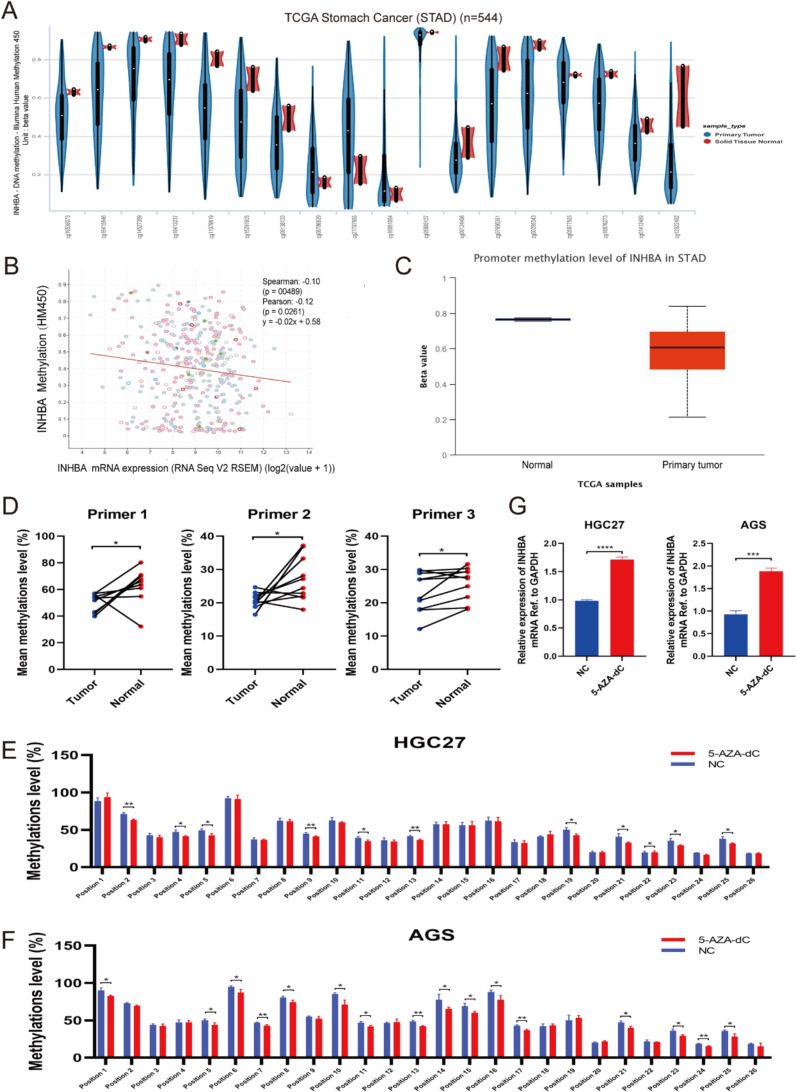
DNA hypomethylation increases INHBA expression and activates TGF-β pathway. **(A)** The methylation level of 18 DNA methylation sites was compared between tumors and normal tissues based on TCGA database; **(B)** Bioinformatics analysis showed a negative correlation between INHBA methylation (HM450) and mRNA expression using TCGA database (The image was generated online using cBioPortal. Spearman’s and Pearson’s correlation tests were utilized, respectively); **(C)** The promoter methylation level of INHBA was compared between tumors and normal tissues based on TCGA database; **(D)** The methylation levels in the first exon region of INHBA were compared between tumors and normal tissues based on three independent primers. **E-F**. After treatment with 5-AZA-dC, the methylation levels of most positions in the CpG islands in the first exon region of INHBA decreased in HGC27 (**E**) and AGS cells (**F**). **G**. The expression of INHBA increased after 5-AZA-dC treatment (*** *p*<0.001, **** *p*<0.0001; t-test). The data are presented as means ± SD

Bioinformatics analysis of TCGA data revealed lower DNA methylation levels at most methylation sites in tumors compared to normal tissues (Fig. 7A). Furthermore, the expression of INHBA displayed a negative correlation with DNA methylation levels (Fig. 7B). These findings suggested that INHBA may be regulated by DNA methylation. About 70% of genes possess CpG islands in their promoter regions, while the remaining genes exhibit CpG islands in other positions [28], such as the first exon region. However, despite the tendency for the level of DNA methylation in the promoter region of INHBA to be lower in tumor tissue compared to normal tissue, this difference did not achieve statistical significance (Fig. 7C). Furthermore, when predicting CpG islands in INHBA, it was observed that INHBA lacked obvious CpG islands in its promoter and enhancer regions, but CpG islands were present in the first exon region (Supplementary Table 5), which piqued our interest. These findings suggested that DNA methylation modification might occur in the first exon region to regulate the expression of INHBA. Therefore, we further assessed the DNA methylation status of the first exon region of INHBA. The impact of DNA methylation status on gene expression in this region has not been extensively investigated, adding an element of novelty to our study. Consequently, we assessed the DNA methylation levels in the first exon region of INHBA.

Pyrosequencing was employed, and three independent primers were utilized to compare DNA methylation differences between tumors and normal tissues. The results demonstrated lower DNA methylation levels in tumors compared to those in normal tissues (Fig. 7D). We also compared the changes in methylation levels of CpG island positions within the first exon region after treatment with a DNA methylation inhibitor (5-AZA-dC) using pyrosequencing. The results showed that after treatment with 5-AZA-dC, the methylation levels of most positions within the CpG islands decreased (Fig. 7E, F). In addition, treatment with 5-AZA-dC resulted in an increased expression of INHBA (Fig. 7G and Supplementary Fig. 2B). We hypothesized that alterations in the methylation levels of the first exon region may lead to changes in INHBA expression. Moreover, the findings of Qiu et al. [6] and Chen et al. [5] indicated that loss of INHBA deactivated the TGF-β pathway.

### Rescue of INHBA silencing by DNA methylation inhibitor

**Fig. 8:**
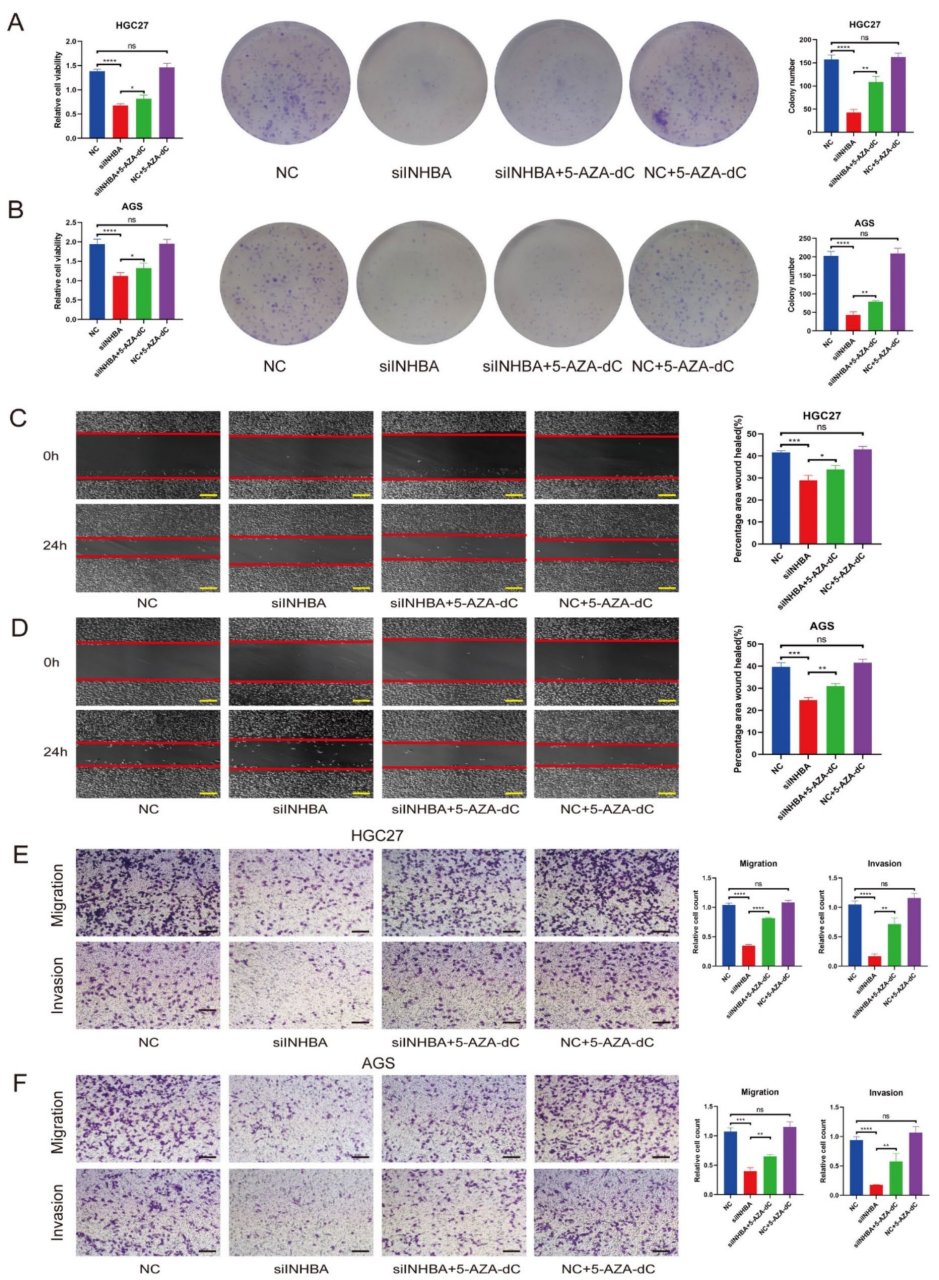
DNA methylation inhibitor could partial rescue the phenotype induced by INHBA-knockdown. **A-B.** Rescue experiments (CCK-8 and colony formation assays) were conducted to assess the impact of a DNA methylation inhibitor on the growth of INHBA-knockdown STAD cells (* *p*<0.05, ** *p*<0.01, *** *p*<0.001; *t*-test); **C-F.** Rescue experiments (Wound healing assays and Transwell assays) were conducted to test the effect of a DNA methylation inhibitor on migration / invasion of INHBA-knockdown STAD cells (* *p*<0.05, ** *p*<0.01, *** *p*<0.001, **** *p*<0.0001; *t*-test)

To investigate the functional rescue of INHBA silencing, we conducted several assays and observed that silencing of INHBA led to a decrease in proliferation ability, which could be partially reversed with treatment with a DNA methylation inhibitor (5-AZA-dC) (Fig. 8A and B, and Supplementary Fig. 3). Additionally, treatment with 5-AZA-dC rescued the reduced migration and invasion capacity of GC cells (Fig. 8C-F). We conducted functional experiments to compare the growth and migration/invasion capability of gastric cancer cells between the negative control (NC) and NC + 5-AZA-dC groups. However, we did not observe any statistically significant differences between the two groups in growth or migration/invasion capability. This can be interpreted as 5-AZA-dC being a broad-spectrum DNA methylation inhibitor that has the potential to increase the expression of oncogenes, such as INHBA, as well as tumor suppressor genes. These findings collectively suggest that DNA hypomethylation of INHBA contributes to the development of GC.

## Discussion

In this work, we conducted a comprehensive analysis of the gene expression and clinical impact of inhibin beta family genes in GC. Among these genes, INHBA emerged as a key candidate for further investigation. By constructing a prediction model that incorporating INHBA expression and clinicopathologic features, we observed improved prognostic accuracy in predicting the outcomes of STAD, suggesting that INHBA may serve as a novel biomarker for outcome prediction of GC Subsequently, in vitro experiments revealed that INHBA plays a significant role in promoting the growth, migration, and invasion capabilities of GC cells. Mechanistically, we found that DNA hypomethylation contributes to increased INHBA expression and activation of the TGF-β pathway. Furthermore, we explored the relationship between INHBA and markers associated with immune checkpoint inhibitors (ICIs), as well as the impact on immune cell infiltration and chemosensitivity, aiming to provide valuable insights for the clinical application of immunotherapy and chemotherapy.

In clinical practice, TNM staging is commonly used to predict patient outcomes. However, numerous studies have highlighted the limitations of relying solely on TNM staging and demonstrated that integrating gene expression data and clinical characteristics significantly enhances outcome predictions [29–31]. We crafted a predictive model that integrates INHBA expression, age, and TNM staging, achieving superior accuracy in forecasting the outcomes of STAD compared to relying solely on TNM staging. For instance, a 55-year-old individual with a low score (0 points) but high INHBA expression (approximating 26 points) and TNM stage III (around 60 points) exhibited a 3-year OS rate of approximately 46% and a 5-year OS rate of approximately 34%. The utilization of our novel nomogram incorporating INHBA enhances the clinical applicability of this biomarker.

Epigenetic modifications, including DNA methylation and histone modifications, play a crucial role in the regulation of gene expression, particularly in gene silencing processes [32, 33]. The promoter region is recognized as a key site for DNA methylation and has been extensively investigated [34]. It is widely acknowledged that epigenetic modifications within the CpG island promoter region can lead to gene inactivation [35]. However, the mechanisms underlying the impact of epigenetic regulation in the first exon on oncogenes remain poorly understood. Our bioinformatics analysis revealed no significant differences in the DNA methylation status of the INHBA promoter region between tumor and normal tissues. Nevertheless, we observed an inverse correlation between DNA methylation levels and INHBA expression. Consequently, we focused our investigation on the methylation status of the first exon region. Pyrosequencing analysis demonstrated a low level of DNA methylation level in the first exon region of the tumor samples. Notably, treatment with a DNA methylation inhibitor resulted in increased INHBA expression, rescuing the effects of INHBA silencing. Compared to research on the promoter region, DNA methylation in the first exon is less popular. However, the modifications in the first exon also possesses important biological functions. Brenet et al. [36] discovered that DNA methylation of the first exon is closely associated with transcriptional silencing. Sakamoto et al. [37] demonstrated that DNA methylation in the exon 1 region complexly regulates Twist1 expression in GC cells. Our study suggests that DNA hypomethylation in the first exon region may represent a novel epigenetic modification mechanism regulating INHBA gene expression. These findings contribute to the understanding of INHBA’s epigenetic modifications, which have not been previously reported.

In recent years, immunotherapy has emerged as a revolutionary treatment option for advanced GC. However, due to tumor heterogeneity and immune escape, a significant proportion of GC patients do not respond to immunotherapy. To identify potential beneficiaries of immunotherapy, various factors such as microsatellite instability status, tumor mutational burden, and PD-L1 expression have been proposed. However, their low positive rates limit their effectiveness in meeting the needs of patients. In our study, we observed significantly elevated expression of ICIs-related markers in the high INHBA expression group, along with a correlation between suppressive immune cell infiltration and INHBA expression. These findings suggest that INHBA expression could potentially serve as a biomarker for assessing the efficacy of immunotherapy. Aberrant expression of INHBA may expedite GC development by modulating the components of the tumor immune microenvironment. We recommend further validation of this hypothesis through additional basic experiments.

In the advanced stages of gastric cancer, chemotherapy represents the primary treatment modality aimed at improving the patient’s quality of life, alleviating symptoms, and extending OS. However, the efficacy of chemotherapy is often limited, accompanied by various side effects. Gene expression alterations have been shown in numerous studies to potentially influence a patient’s response to chemotherapy [38, 39]. Therefore, to minimize adverse effects and optimize therapeutic outcomes, it is crucial to identify appropriate drug combinations and dosages. In this study, we investigated the impact of INHBA expression on chemosensitivity and found that patients in the INHBA-high group were more likely to benefit from cisplatin, docetaxel, and camptothecin. Thus, we propose that combining cisplatin with docetaxel or irinotecan may yield superior results for patients with high INHBA expression.

This study has several limitations that should be acknowledged. Firstly, the absence of animal experiments represents a major limitation in the basic experimental component of our study. Although we initially employed the BGC823 cell line for both cell function and subcutaneous xenograft experiments, yielding positive results (data not shown), we subsequently identified contamination of BCG823 with Hela cells, rendering it an inappropriate cell line for studying GC in this manuscript. Moreover, we discovered that Chen et al. [5] had already conducted subcutaneous xenograft using INHBA stable knockdown SGC7901 cells. Due to personal reasons, the authors did not repeat the animal experiment with other GC cell lines. Secondly, the lack of sufficient external validation using independent data is a notable limitation of our study. Thirdly, we believed that conducting independent RNA-seq or proteome analysis on the INHBA knockdown or overexpression GC cell line would greatly contribute to strengthening our conclusion. Unfortunately, practical limitations prevent us from carrying out these analyses at present. Consequently, we recognize the necessity of future studies that can incorporate additional techniques to more comprehensively address the questions raised, such as single-cell sequencing and spatial transcriptomics. Fourthly, 5-AZA-dC is a broad-spectrum inhibitor of DNA methylation. The rescue of biological function in INHBA silencing by 5-AZA-dC may be attributed to its activation of other oncogenes or transcription factors involved in INHBA regulation. Changes in DNA methylation levels may represent just one method of regulating INHBA. Fifthly, the associations between INHBA expression and immune status, as well as chemotherapy sensitivity, were solely analyzed based on bioinformatics data. It is imperative to conduct additional basic experimental research to further elucidate the impact of INHBA on the immune microenvironment and chemotherapy sensitivity. Overall, it is recommended to perform further in vivo experiments in subsequent studies to assess the effects of INHBA on the proliferation and migration of gastric cancer (GC) cells, as well as to verify the regulatory role of DNA hypomethylation in the first exon region on INHBA expression. Additionally, both in vivo and in vitro drug resistance experiments are recommended to further investigate the influence of INHBA expression on the sensitivity of GC cells to the most commonly used platinum-based drugs or taxanes.

## Conclusions

Our study demonstrates the oncogenic role of INHBA in GC. We have identified that DNA hypomethylation contributes to increased INHBA expression and activation of the TGF-β pathway. We emphasize the potential of INHBA as a novel clinical biomarker for predicting patient prognosis, as well as for predicting sensitivity to immunotherapy and chemotherapy.

## Electronic supplementary material

Below is the link to the electronic supplementary material.

Supplementary Material 1

Supplementary Material 2

Supplementary Material 3

Supplementary Material 4

Supplementary Material 5

Supplementary Material 6

Supplementary Material 7

## Data Availability

No datasets were generated or analysed during the current study.

## References

[1] Bray F, Ferlay J, Soerjomataram I, et al. Global cancer statistics 2018: GLOBOCAN estimates of incidence and mortality worldwide for 36 cancers in 185 countries. CA Cancer J Clin. 2018;68(6):394–424. 10.3322/caac.21492.PMID. 30207593 10.3322/caac.21492

[2] Joshi SS, Badgwell BD. Current treatment and recent progress in gastric cancer. CA Cancer J Clin. 2021;71(3):264–79. 10.3322/caac.21657.PMID. 33592120 10.3322/caac.21657 PMC9927927

[3] Zhu Y, Zhu X, Wei X, et al. HER2-targeted therapies in gastric cancer. Biochim et Biophys acta Reviews cancer. 2021;1876(1):188549. 10.1016/j.bbcan.2021.188549. PMID: 33894300. 10.1016/j.bbcan.2021.188549 33894300

[4] Derynck R, Turley SJ, Akhurst RJ. TGFβ biology in cancer progression and immunotherapy. Nat Rev Clin Oncol. 2021; 18(1):9–3410.1038/s41571-020-0403-1.PMID: 32710082. 10.1038/s41571-020-0403-1 PMC9721352 32710082

[5] Chen ZL, Qin L, Peng XB, et al. INHBA gene silencing inhibits gastric cancer cell migration and invasion by impeding activation of the TGF-β signaling pathway. J Cell Physiol. 2019;234(10):18065–74. 10.1002/jcp.28439.PMID. 30963572 10.1002/jcp.28439

[6] Qiu S, Li B, Xia Y, et al. CircTHBS1 drives gastric cancer progression by increasing INHBA mRNA expression and stability in a ceRNA- and RBP-dependent manner. Cell Death Dis. 2022;13(3):266. 10.1038/s41419-022-04720-0.PMID. 35338119 10.1038/s41419-022-04720-0 PMC8949653

[7] Kumar V, Ramnarayanan K, Sundar R, et al. Single-cell atlas of Lineage States, Tumor Microenvironment, and subtype-specific expression programs in gastric Cancer. Cancer Discov. 2022;12(3):670–91. 10.1158/2159-8290.Cd-21-0683.PMID. 34642171 10.1158/2159-8290.CD-21-0683 PMC9394383

[8] Ren J, Lu P, Zhou X, et al. Genome-scale methylation analysis of circulating cell-free DNA in gastric Cancer patients. Clin Chem. 2022;68(2):354–64. 10.1093/clinchem/hvab204.PMID. 34791072 10.1093/clinchem/hvab204

[9] Nishiyama A, Nakanishi M. Navigating the DNA methylation landscape of cancer. Trends Genet. 2021;37(11):1012–27. 10.1016/j.tig.2021.05.002.PMID. 34120771 10.1016/j.tig.2021.05.002

[10] Győrffy B. Integrated analysis of public datasets for the discovery and validation of survival-associated genes in solid tumors. Innovation (Cambridge (Mass)). 2024; 5(3):100625.10.1016/j.xinn.2024.100625.PMID: 38706955. 10.1016/j.xinn.2024.100625 PMC11066458 38706955

[11] Chandrashekar DS, Karthikeyan SK, Korla PK, et al. UALCAN: an update to the integrated cancer data analysis platform. Neoplasia (New York NY). 2022;25:18–27. 10.1016/j.neo.2022.01.001.PMID. 10.1016/j.neo.2022.01.001 PMC8788199 35078134

[12] Chandrashekar DS, Bashel B, Balasubramanya SAH, et al. UALCAN: a portal for facilitating Tumor Subgroup Gene expression and survival analyses. Neoplasia (New York NY). 2017;19(8):649–58. 10.1016/j.neo.2017.05.002.PMID. 10.1016/j.neo.2017.05.002 PMC5516091 28732212

[13] Cerami E, Gao J, Dogrusoz U, et al. The cBio cancer genomics portal: an open platform for exploring multidimensional cancer genomics data. Cancer Discov. 2012;2(5):401–4. 10.1158/2159-8290.Cd-12-0095.PMID. 22588877 10.1158/2159-8290.CD-12-0095 PMC3956037

[14] Gao J, Aksoy BA, Dogrusoz U, et al. Integrative analysis of complex cancer genomics and clinical profiles using the cBioPortal. Sci Signal. 2013;6(269):pl1. 10.1126/scisignal.2004088.PMID. 23550210 10.1126/scisignal.2004088 PMC4160307

[15] Goldman MJ, Craft B, Hastie M, et al. Visualizing and interpreting cancer genomics data via the Xena platform. Nat Biotechnol. 2020;38(6):675–8. 10.1038/s41587-020-0546-8.PMID. 32444850 10.1038/s41587-020-0546-8 PMC7386072

[16] Ru B, Wong CN, Tong Y, et al. TISIDB: an integrated repository portal for tumor-immune system interactions. Bioinf (Oxford England). 2019;35(20):4200–2. 10.1093/bioinformatics/btz210.PMID. 10.1093/bioinformatics/btz210 30903160

[17] Li T, Fan J, Wang B, et al. TIMER: a web server for Comprehensive Analysis of Tumor-infiltrating Immune cells. Cancer Res. 2017;77(21):e108. 10.1158/0008-5472.Can-17-0307.PMID. 29092952 10.1158/0008-5472.CAN-17-0307 PMC6042652

[18] Yang W, Soares J, Greninger P, et al. Genomics of Drug Sensitivity in Cancer (GDSC): a resource for therapeutic biomarker discovery in cancer cells. Nucleic Acids Res. 2013;41(Database issue):D955–61. 10.1093/nar/gks1111.PMID. 23180760 10.1093/nar/gks1111 PMC3531057

[19] Garnett MJ, Edelman EJ, Heidorn SJ, et al. Systematic identification of genomic markers of drug sensitivity in cancer cells. Nature. 2012;483(7391):570–5. 10.1038/nature11005.PMID. 22460902 10.1038/nature11005 PMC3349233

[20] Cristescu R, Lee J, Nebozhyn M, et al. Molecular analysis of gastric cancer identifies subtypes associated with distinct clinical outcomes. Nat Med. 2015;21(5):449–56. 10.1038/nm.3850.PMID. 25894828 10.1038/nm.3850

[21] Zheng Y, Heagerty PJ. Semiparametric estimation of time-dependent ROC curves for longitudinal marker data. Biostatistics (Oxford England). 2004;5(4):615–32. 10.1093/biostatistics/kxh013.PMID. 15475423 10.1093/biostatistics/kxh013

[22] Foucher Y, Danger R. Time dependent ROC curves for the estimation of true prognostic capacity of microarray data. Stat Appl Genet Mol Biol. 2012;11(6):23183763. 10.1515/1544-6115.1815.PMID. Article 1. 10.1515/1544-6115.1815 23183763

[23] Steyerberg EW, Vickers AJ. Decision curve analysis: a discussion. Medical decision making: an international journal of the Society for Medical Decision Making. 2008; 28(1):146-9.10.1177/0272989x07312725.PMID: 18263565. 10.1177/0272989X07312725 PMC2577563 18263565

[24] Tost J, Gut IG. DNA methylation analysis by pyrosequencing. Nat Protoc. 2007;2(9):2265–75. 10.1038/nprot.2007.314.PMID. 17853883 10.1038/nprot.2007.314

[25] Greville G, Llop E, Howard J, et al. 5-AZA-dC induces epigenetic changes associated with modified glycosylation of secreted glycoproteins and increased EMT and migration in chemo-sensitive cancer cells. Clin Epigenetics. 2021;13(1):34. 10.1186/s13148-021-01015-7. PMID: 33579350. 33579350 10.1186/s13148-021-01015-7 PMC7881483

[26] Cai X, Chen Y, Man D, et al. RBM15 promotes hepatocellular carcinoma progression by regulating N6-methyladenosine modification of YES1 mRNA in an IGF2BP1-dependent manner. Cell Death Discov. 2021;7(1):315. 10.1038/s41420-021-00703-w.PMID. 34707107 10.1038/s41420-021-00703-w PMC8551180

[27] Li X, Gu M, Hu Q, et al. Development and validation of metabolic models for predicting survival and immune status of hepatocellular carcinoma patients. Adv Clin Exp Med. 2023;37166013. 10.17219/acem/162819.PMID. 10.17219/acem/162819 37166013

[28] Deaton AM, Bird A. CpG islands and the regulation of transcription. Genes Dev. 2011;25(10):1010–22. 10.1101/gad.2037511.PMID. 21576262 10.1101/gad.2037511 PMC3093116

[29] Li X, Yu W, Liang C et al.,*. INHBA is a prognostic predictor for patients with colon adenocarcinoma. BMC cancer. 2020; 20(1):305.*10.1186/s12885-020-06743-2.*PMID: 32293338.* 10.1186/s12885-020-06743-2 PMC7161248 32293338

[30] Teichgraeber DC, Guirguis MS, Whitman GJ. Breast Cancer Staging: Updates in the AJCC Cancer Staging Manual, 8th Edition, and Current Challenges for Radiologists, From the AJR Special Series on Cancer Staging. AJR American journal of roentgenology. 2021; 217(2):278 – 90.10.2214/ajr.20.25223.PMID: 33594908. 10.2214/AJR.20.25223 33594908

[31] Adsay NV, Bagci P, Tajiri T, et al. Pathologic staging of pancreatic, ampullary, biliary, and gallbladder cancers: pitfalls and practical limitations of the current AJCC/UICC TNM staging system and opportunities for improvement. Semin Diagn Pathol. 2012;29(3):127–41. 10.1053/j.semdp.2012.08.010.PMID. 23062420 10.1053/j.semdp.2012.08.010

[32] Stoll S, Wang C, Qiu H. DNA methylation and histone modification in hypertension. Int J Mol Sci. 2018;19(4):1174. 10.3390/ijms19041174.PMID. 29649151 10.3390/ijms19041174 PMC5979462

[33] Cai X, Liang C, Zhang M, et al. N6-methyladenosine modification and metabolic reprogramming of digestive system malignancies. Cancer Lett. 2022;35798087. 10.1016/j.canlet.2022.215815.PMID. 10.1016/j.canlet.2022.215815 35798087

[34] Spagnol LW, Polettini J, Silveira DA, et al. P16 gene promoter methylation is associated with oncogenesis and progression of gastric carcinomas: a systematic review and meta-analysis. Crit Rev Oncol/Hematol. 2022;36270449. 10.1016/j.critrevonc.2022.103843.PMID. 10.1016/j.critrevonc.2022.103843 36270449

[35] Angeloni A, Bogdanovic O. Enhancer DNA methylation: implications for gene regulation. Essays Biochem. 2019;63(6):707–15. 10.1042/ebc20190030.PMID. 31551326 10.1042/EBC20190030

[36] Brenet F, Moh M, Funk P, et al. DNA methylation of the first exon is tightly linked to transcriptional silencing. PLoS ONE. 2011;6(1):e14524. 10.1371/journal.pone.0014524.PMID. 21267076 10.1371/journal.pone.0014524 PMC3022582

[37] Sakamoto A, Akiyama Y, Shimada S, et al. DNA methylation in the exon 1 region and complex regulation of Twist1 expression in gastric Cancer cells. PLoS ONE. 2015;10(12):e0145630. 10.1371/journal.pone.0145630.PMID. 26695186 10.1371/journal.pone.0145630 PMC4687923

[38] Zhong T, Zhang W, Guo H, et al. The regulatory and modulatory roles of TRP family channels in malignant tumors and relevant therapeutic strategies. Acta Pharm Sinica B. 2022;12(4):1761–80. 10.1016/j.apsb.2021.11.001.PMID. 10.1016/j.apsb.2021.11.001 PMC9279634 35847486

[39] Konoshenko M, Lansukhay Y, Krasilnikov S, et al. MicroRNAs as predictors of lung-Cancer resistance and sensitivity to Cisplatin. Int J Mol Sci. 2022;23(14):7594. 10.3390/ijms23147594.PMID. 35886942 10.3390/ijms23147594 PMC9321818

