# Supplementary material for: DNA hypomethylation of INHBA promotes tumor progression and predicts prognosis and immune status of gastric cancer
Source: Hereditas. 2024 Nov 14;161:45. doi: 10.1186/s41065-024-00347-7 (PMC11562481; doi:10.1186/s41065-024-00347-7)
Supplement: Supplementary file 1 — Supplementary Material 1 [file 41065_2024_347_MOESM1_ESM.docx]

Supplementary Table 1. Primers employed in this study for pyrosequencing

| Primer | Sequence (5’ to 3’) |
| --- | --- |
| 1.INHBA-1F(230bp) | AGGGATAGTTGTTGTTATAGGTAAAT |
| 1.INHBA-1R(Q48) | CCTATCTTACTACTACTAACTCCAATAC |
| 1.INHBA-1S | GTTATAGGTAAATATTATTATAGGG |
| 2.INHBA-2F(192bp) | TGGTAGAGGGTTATTTTGTTAGTGTAG |
| 2.INHBA-2R(Q48) | ATCTCTAAACCCAACCCTCAATA |
| 2.INHBA-2S | GGTTATTTTGTTAGTGTAGT |
| 3.INHBA-3F(255bp) | GTTTTTGTTGGTGTTTAGGGTAAAAATAG |
| 3.INHBA-3R(Q48) | CCAAAATCTCCTTCTCCTTCCTTAAT |
| 3.INHBA-3S | GTGTTTAGGGTAAAAATAGAG |
