# Supplementary material for: DNA hypomethylation of INHBA promotes tumor progression and predicts prognosis and immune status of gastric cancer
Source: Hereditas. 2024 Nov 14;161:45. doi: 10.1186/s41065-024-00347-7 (PMC11562481; doi:10.1186/s41065-024-00347-7)
Supplement: Supplementary file 2 — Supplementary Material 2 [file 41065_2024_347_MOESM2_ESM.docx]

Supplementary Table 2. Primers employed in this study for RT-qPCR

| Gene symbol | Forward primer | Reverse primer |
| --- | --- | --- |
| GAPDH | GGAGCGAGATCCCTCCAAAAT | GGCTGTTGTCATACTTCTCATGG |
| INHBA | CCTCCCAAAGGATGTACCCAA | CTCTATCTCCACATACCCGTTCT |
