# Supplementary material for: DNA hypomethylation of INHBA promotes tumor progression and predicts prognosis and immune status of gastric cancer
Source: Hereditas. 2024 Nov 14;161:45. doi: 10.1186/s41065-024-00347-7 (PMC11562481; doi:10.1186/s41065-024-00347-7)
Supplement: Supplementary file 3 — Supplementary Material 3 [file 41065_2024_347_MOESM3_ESM.docx]

Supplementary Table 3. Antibody employed in this study

| Antibody | Source | Identifier |
| --- | --- | --- |
| GAPDH | Proteintech | Cat#60004-1-Ig |
| INHBA | Proteintech | Cat#60352-1-Ig |
