# Supplementary material for: DNA hypomethylation of INHBA promotes tumor progression and predicts prognosis and immune status of gastric cancer
Source: Hereditas. 2024 Nov 14;161:45. doi: 10.1186/s41065-024-00347-7 (PMC11562481; doi:10.1186/s41065-024-00347-7)
Supplement: Supplementary file 4 — Supplementary Material 4 [file 41065_2024_347_MOESM4_ESM.docx]

Supplementary Table 4. RNA interference employed in this study

| Gene symbol | Targeted sequence |
| --- | --- |
| siINHBA#1 | AUAAGUUCAUUCAUUUCUGCCTT |
| siINHBA#2 | UUUCUGAUCGCGUUCAGAAGCTT |
