# Supplementary material for: DNA hypomethylation of INHBA promotes tumor progression and predicts prognosis and immune status of gastric cancer
Source: Hereditas. 2024 Nov 14;161:45. doi: 10.1186/s41065-024-00347-7 (PMC11562481; doi:10.1186/s41065-024-00347-7)
Supplement: Supplementary file 5 — Supplementary Material 5 [file 41065_2024_347_MOESM5_ESM.docx]

Supplementary Table 5. The prediction of CpG Islands Position in INHBA Sequence

| The sequence of INHBA |
| --- |
| ACCTCAAGTGATCCACCTGCTTCAGTCTCCCAAAGTGCTGGGATTACAGGCATGACCCAC**CGCG**TCTGGCATAAATATGTCCACATTTTTAAATCCAGTGATTCCACTTCTAGGAACTTATCTCAAGGAAATAATAAGAGATGCATTTAAATACATATAAGGATGGATGCAATACTTATACTTACAAAATTTGGGAAATGACTTCAATATACAATAATTGTGGACTGGTTTAAAAATAAAGACACACCTATTTGAAAGGTTACTCCCCAGTCATTAATATTTTCAAGAAATAATTTGACTATTGAAAATGCTCAACATACAGTGATAGGTGAAAAATATCAGTATACAGGATTATATTACAGTAGGATTGCAATTTTTTTTTTTACACATACACAGAAATTTTAATAA**CG**ATTATTTCTGGAAGGGATGATTACATGTGATTTACATTTTTTGTTCTG**CG**TCTTCAACATTTTATACAAATAATATG**CG**TCTTTTTTAAGCTTAGGATGGATGTCATTTAAAAAGCAACAATTTTTGGTATTGGAAGCAGACAGATCA**CG**GAGAAGGTGCCCAATTGCTACAGGGTTTCTTTCTACACAGGGTTTCTTACTATTCAGATGCACCAGAAGGGGG**CG**CCTCAGCCTCTTCC**CG**CTGACAGACAGCTGGATCACCTGGTAAGTCTGGAATGTTCAGGTCTGCAGGAATCACTTTCCCT**CG**T**CG**TTA**CG**CTTCTTTTCTGGGTCTCTCAGAAATTTCCATAGAAGAAGGATATGGAAATTTGAATGCCACCATGAGAGTTTTCTCTAGTCACATGATTAACAGTCTGTGCTGGTGATGTGTCCTTTGGTCAACACTCTTCTACCTGGGCAGCTGCTGCAAATCAGGAGTGCCTCTCCATCAGGAAAAGACAGAAACAAAACAGACTCTACC**CG**ATGACTTTGCCTTAAT**CG**CAACAAACAGGTTCATGGAAATGGCCAAATTCACAACTATAACTTAGGAGGTTGACTCAGCCACTAACTAATTGTACCTGAATCAGACTTTGTTTCTTAAAAAGTTATGTGCTGTTCTTCATGGTACCAGTTAGGGGGCAGCAGATAGCCTGGCATCTTTTGTTGTCTAAAGGAAGCTTCCAATTAGGAGATCCTAAAGTTTCTATGTATTATTTTATTTATCATGTGACATGGTCTGACAGTTCCATCCTG**CG**CCAGCTGTTCATTTTGGAAGCCAAACCTCTCACCTAGTAGAGTGCTTATAATTCACTACTGAGCAAAGGTGAACCCAAATAATAAAGTATCCTATATAATTACTGCACTCTGCATCAGCCATTTCAGTTTCATCATTATCTAAATATTA**CG**AAGCCCTAGCCTTCTCTTTTGGTCTATCATTCTGTCTCTCACACA**CG**CCTAAGTAAACTTTATTTTGACTCCTTAAAATACCCTCAGGATGAGAATGCAAGTAATGGGATGAGTGTAGGCATCTATAAAATGGCTTCTGCAAAGACCTCAAGATGCTAGTTTAAGAGGATGAGGCATAGCCAGCCTGT  GGTAAAAACATTTGCTTTCCTGATGCATGCAACATTGTGGAGATGCTACTTTTGTGTGTGGGGTGTGTGTGTTTGTGTGTGTGTGTGTGTGTGTGTGTGTAGCTACTGAACACTGAGAATTTTGTCTATTCAAAAAGAAACAACAGAAACATGATGCTGAGCTCTTGAGGATCCTTGAACCAATGTCTTCTTTTGGTTTTGATTA**CG**TACCTATTTTAGTTTCCAGAAGCTCCTCATGGGATAAAGAGTAGTAGGTGTGGAAACCAGATCACAGAGAGGTAGGCTGACTTGTAACTTTCCAGCCAGGGACAGAGCCCCTGTGTGTAGTAGGGTCTTTTCAGTACCAGTCTACCCTGGTGGCTTGTCACAGAGCATTTGCCCATCTTTCCAAGGCTCTACAAAATAGGATGCAGGGAATTCAGGTCAGCCAATAAAGCTTTTTTAAAAAAGGAAGAGAAATAATACCAGTAAGCAACATTGACTGTGTACCAGCACATTCTATG**CG**GCTCACA**CG**ACTAACTCAGCTATCCCTGACAACAATCCTATCTATGAATATTATTATCCATATTTATTATACTAATTGTGGAACCAAGTCATTTGCCCAGGAGTTTGAAAGTGGTGAATCCTAAATTTGAACCCAGTCAGTCTGACTGCAGGACCCACACATTCAACCACTACCTTTTGCCTCATCTTCAAATGGTGTTTTGCATACTTCAGAGCACTGAGGTGGCTGTGCCCTTGGTCACTGGGAGGCCATACTTGGCTGCACCCATTTGGACTGTCAGGATCTTCTCCAGTCAGAGGCCAC**CG**CCAGGGCCAGAGACAATGGAGAATTATTTAGTATCCAGTGGTTCTAGAAACTGCCTCTGTTTCAAGCCCAGGAAGAAGGCTTTCTGAGGAGGACATTCCTTCTTGGGACAAGGACCCTTAGTGAGATTTGGCCTTTATGCACCTCCATCTGAAAAGCTAAGCTTTTCTCTGGGAATTTGAATTGAAGGTTAGGGATGATTGCTCAGATGGGGAAAGTGTGAAATGGTAATCACTTTCATATGACACT**CG**AGAAATGTTATAATAAAGCTGAGTAAATTGGAGCATGAGTTAGC**CG**GTCCTTTGCCTAATGATGTCACTCACTTTGTGCTTCCTCCAGCCTATGATTAATAAAAGAGGGGTTATGACAGTAACTCACTCCTAGTGGGCAGCAGTGA**CG**TCAGCAGCAGCTGAGCAAACTGACTACATTTAGTTGTG**CG**TGTGTATGTGGGCATTTTTTTTTCCTGTGACTAACATGAAGATAACAGCATCTAATAAAACAAAAGCTCCTTGAAATTTGGGAAACTTATTGGGTGAACTCTTTTCTACAGGGAGCACCATTTTAATTATTCTCAGCTCATAGTTAAGTGATGTTGGAGTTGGCCAAAATTACCTTGTTGGCTTTCTGCCCCTGTTTCATTAAGGGATCACAAATGAACCCCTGGCCTTTTGGAGAGAGGTTTTCTGGAGTGTTTTTTTCCTTCTACTATAGAGGCTCCTCCCTCATTCATTATACTCTTCCATCTTTTAAGAGGAACTCAATGAGTAGAAGAGGGATTGTAGCCATAAGTTATTTTTTGAAAGTTTAAAGCTGGTTGTAGAATTCAGTGATTCCATTTTAAACTTTGTAAGAATATTGTGCTTAATTACAGTTTGCTTTTCCAATCTAAAGTGACAGAAGTCCTACTTGCTATTTCACCTTTGCAAACTTGACCTTCCTAAAATTAGAGTGGGCCACCTGGAAATATTTACTCACCTAGCTGAGCTGCTGCCAGTGTCCA**CGCG**AGTCCACAGGACT**CG**TGAACAGCAGCACTCAGCCCATGCCTTGGAGCTGCTCCCACAGAGGGCAGAACCTTTGGACTCTATTAGGACA**CG**AGGGAGGCAGCAATTCACTGATTTAAAAAAAATACACACAGAACTGCCCCCAGGCTTTTCTGT**CG**GGCTTTTTCCCTCCAAGCAG**CG**ATTTGGGAACAGGTTGTGGGGGGATCTTTGTGGACAGGCAAAAGTGGATGCAAATCAAGATTGCTGCTCTCTTATCAGTCCAATGGAATCTA**CG**TGCTAAACCACCATGTCTTCAAGTCACCAATGGAACTTATTACCCAAGGGGATCCAGGTGAGAAAAATGATTACAACAATCTGTTCCGCATTCCTTGGGGGGTATTTTTCAATAATCTTCTGAGGCAATAGGAAGATAAATTGTTGAAGCAGATGTTTCCTGATTGGGTTAGAACTGAGTCATGATAAACAATTTTGAAATATATATTGGTCAGGAAGAATGTTGAGGGCATATTTCTTCAGGTTGAGTTAGTCTGGGTTTTTTTCAATATATGTTTTTATATAGTGGTTGTAAATGAGTATTTCTTTCAATATTGAATTTTTTTTACATGTACTACATGGTTACTTCTTGGGAAGAAGTCCAGGCATTTCTGAATCATTTGAAAGTTGATTGAGGCCTTTGGTTCCAAAATGACCATTTACCTGAGGAGACAGTGATTCACA**CG**TTGGAAGAGATGATA**CG**G**CG**TCAGAGTGTCTAAGACATTCCCCTCATGGCTCCCTTCATCTTAAAATGATCTCTGACCTACCCTTTCCTTTCTGGATGACCCATTTTACTCAGGAGCTAAGTGATGAACCAGGGAAGTAAGCACTCACTGTATGGACAACAGGATTTCAGTTCTAAGCTAGTTTTATCCAGGCAACTTCCCTCTTGTGCATTTCACCAATACTTGTTGATTACTCTTA**CG**ATTTTCCATGAGATGGAATCCTTCACAATAGTTTCCCCAGTCCTCACAGGAGAGCAACACCTGGAAGGGCATGGCCTTCCTTTAATAGACACACCACTTCCTTTTACAAAGGAAGTTTGGAGAATATAATGTTGCTTGCAACAGCCATTAGGATTTAGCAAAACACTGTTTTATCTCTTTGGATATTGATTGTATGTCATTCCCCTAAGGTATTAAACAAATAAAATCAAGCTACATCATATTTGTTTGCTCTAGGAGAAAGGCATGGTTATCCTA**CG**TTTGTGAATTTTACTTCAGGGAATTCTCTATTGCATGGATTTATCTGATACTGAATTGCTAAAATGAACAACTCAATCCTTTATTCATTTACTCTTTTGCATGAGTCTCTTGTCTCCTAACAACTTGGGTGTGGGCCAGCCCATCTCATTTTGTTGCTGGTCACCAAGATTCTGCTTTAGGTCAGAACAAAAAGCCAGCAGGAAAATGTATTGAGACCATA**CG**TGAAAGTTGGTTGGAACAA**CG**AGGTTGCCCAAGAGGAACATTGTATCTGTAGTCTCCAGTAATCAGAAAACATAAGATGCAAAAGGAAGCAGAATCACTTGAATTATTAGTATTTTGTAGAGCTGCAATTTTTTTTTTTTTTTGGTTTCCAAGGCCTCTTGGAGATGGATAGGGAGAAGGTTGAGTGAAACTTTACAATTATAATATGAAATCAGGGTATCTCAACTGCAGAACAATGAACACTGTAGGCCAGGTAATTTCTTGTTGTTGGGGGCTGTCCTGTGCATGGTAGGATATTCAGCAGCATCCCTGGGCCCTACCCACTAATGATCAGTAGAACCTCCCTCCCAGCTGTAACAACCAAAATGTCTTCAAACATGGCCAGATGTTCCC**CG**GGGAGCAAAAT**CG**CCC**CG**GTTGAGAACCACTGCTGTAGAATGAAGAGTAATAGGTTACTTACTGGGTTTTCTTCCTCCTTCTCTTTTCCCTAATTGTTTTAGTTCTAGTATCTGGGTTCCCTTGGTTCAGAAAGCAGCATTTCCAAATAATCTAAAAACAGAGTGCTCTTACATAGCAGAAGGCCTTGTGGGATATTCTGGTGGTTCTGAAAGAAATGTGAAGCTTA**CG**AGTCAAGTTTCAGATTAACCATTTCTGACCCTGTTGTTCTCAATGAATTTCAGGGGACTTTTTAATGCTTTCCTCTCTCTCTCTGTTT**CG**ATCTATTCATTCATTGATTTAACTTAAAAGCAAAACAGCTTCCCAGTTCAATTTTGAA**CG**TGAAA**CG**CTGATTCTCCTTTGTCCCAAATCTTTCTACCTAACATTCAGCCCAAGCTATGTACAGGCTG**CG**GCTGTCCAGGAAACAGGCAGGGTTTGCCAATCCCATGTT**CG**ATTTAAACCATTTGATGAGACCCTTCATCACAGACTCT**CG**CAGTCTCCCTGCAGCAAATAGTACTCTGGATCCTGCCCA**CG**TTCTCTGGATTCTGAGCTGCCTCTGGAGGGAGTCACCCCTCACCAACAAGGCCAGGTTTTC**CG**GCCTGAGCCCCAGAATAACACATCAGCTGCTGGGAACACCTCGAGTCCTTTTGATTTCTCTCCATAGTCA**CG**TTTTATGTT**CG**GTTTCCCAGA**CG**GGATGAACCAGAAATCATGCTTTTTTGGAAACAGATTTTTTCATAGGAAGGAAAGAAAACAC**CG**TGACT**CG**TCAGCACTCTTGTC**CG**TGCCTCTGAACCCCCAGCCTTGTGTCATTTCAGCTGAATCAGAGAACAACATCCCAGTCACCTGCTTGGGG**CG**TT**CGCG**TCTCCTGGCTTGTATTTTTGACTGAATAACAGCAATC**CG**TGAGCAGCAGCTAAAATAATAGAGCCCTGAAAAATGCCCCAACCCCCACTCCCTTTTCTGGTCCTGTCTCCTTGCTCCAGGAGTAGTGAGGA  AAGAGACCAGTTAATAG**CG**TGGATGAAAGCTGAAGGACAGA**CG**GTTTGAGAAGAAAAGTGAACATTCTTGCTTCCTCATCTATGCCAACTTTGAACAGGGACAGCTGCTGCCACAGGTAAACACCACTATAGGGTTGGC**CG**TGGCCCTAAGCTAGGTGTCAC**CG**TAAGCCA**CG**GGCCAGCACA**CG**AA**CG**CTGGCCTCTCCCCCATT**CG**CACAATTGAGCTCTTATCCTTAAA**CG**AACTTTGTTTTTTGACCAGCTCACTAATTGTGATTGAAAACACCAGCTCCCCAGCAATGCACA**CG**CATTGGAGTTAGCAGCAGCAAGACAGGCCCTAATTGCTCCATTTACATAAAAGTAGAAACATACAACTTCTAAATATAAATCTCTGGCACAT**CG**TTCCTTTTGCAATTACAGTAAAATAAGGTACCACAGTTTCCCTCC**CG**GCTCCTTCT**CG**CAGCAAGCTGCTCCAC**CG**GGTT**CG**CTAGTGGCTGCTCCT**CG**AGG**CG**AGAACAGAGCCATGCAAATGCTCCAG**CG**TGGAGTTGGGTTTGTGAAGT**CG**CCAGTAAATCAGGC**CG**CCTCCACTAGGTGAATAA**CGCG**GTGGCAG**CG**GGCCACTTTGCTAGTGCAGT**CG**CTGGAGGAC**CGCG**CTC**CG**CACTCAGAAGGGGGCAGTTGGCTGGTG**CGCG**AG**CG**CTCC**CG**GCT**CG**CCTCTGTC**CGCG**GGGAGCCAGC**CG**GCCCCTC**CG**CAGGTCC**CG**CTCCCTGACATTTGCTGTGGTTTCACTCCCTGGCACTGAGGGCTGGGTTCAGAGATTCCCAGCTGCACTCACTGAACAGTGATTTTTTTCCCCTCCCTGAATTCTTTAGCTGCCAAAAGGGGGGAAAAAATCAAGAGCTGCTCTTAAAAGAAGTTGCCCTTGCTGGTGCTCAGGGTAAAAATAGAGGCTGC**CG**CTTAGAC**CG**GCTTGGCCCTGGCTCCAGGCATCCTG**CG**AGCTGGGCT**CG**AGCAG**CG**GC**CGCG**TTCCGG**CG**TGATCCCTGGAAGCTGCCAGCAGGTGCTGCTCAAGGTACAGTAGCAGGGC**CG**AGAAGC**CG**GGACCCGAAGGGCCTGGGGTG**CG**GGGAAGCTC**CG**GGGGAGGGGGAAGTGAAAGAGAAACCAAACCAAGGAAGGAGAAGGAGATTCTGGTCAGGACTGGGCTGACCAGCTTGTGCCC**CG**GGCCCTTGAGTTC**CG**CAGGGAAAAGCTGATTCCCAGTCTAGAATATTCCACAGAAGCTATTTTAACCTGCCTGCTTCCTCCACCACCCCCACCTTGATTTGCAC**CG**AGGGAGGAGGCATTGCCACTGTGGTGCAGGTGGTTGCAGAGGGGTTGCAGCA**CG**GCTGGTAGGCTGGCCTGGCAGGTGGGCACT**CG**GTGGCATCAGATGGCCAG**CG**GAGTTTCAGAGTCACACTTGTGTTGTGGCTCCTCTGGTCAGAGAGCTGCAGATTCCTGTGACACCCATGTTTAAGAACAGCCACCAGGCTTCTCAGAAATAGTATCACTTATTATTATCTGACAGCTGCACTTGACCCCCACAGGTTCATATCATGGATCCCATTTTATAGATGGGAACACTGAGGCCTGAGTTTACA**CG**GTGAGTGAGCAGAGGAACTGGGATCTGATCTGGGTTTGTTTTGTGCCCCCCACCCCCACCCCA**CG**TCACCTACACACACACACACACACACACACACACACACACACACACACACACTACTTTGTGTGGAGG**CG**GAGGGGATGTTTTCAGGACACTAGGGTTCATGTGTGGGTCAGGGGTGAGTTTAGATGCCCTGAAGGTCATTGACTCCACTAATTCTCAGCAAAAAGGGTGGAGAAGTTTCTTTCTTATAAAGGGTTTGAGTTCACCCAGTGCTAAGCTGAGGGGCAGGTTTGGAGTTCACCATCATTAATCCTATTTCTGTCTT**CG**CCACTCAGATTCACTCTCTTCCTCTACAACCTAATGATTTTTTTCCTACTTCATTTCTGCATATGCAGTTCTTAGAATACTCCTTCCCCCCACCCCCATGCAGCTCAGCACCCACACTTTCTCTGTATCTAGCCAAGAGAGACCCTTTTACACAGAAAACTTCTGGTGTCCTAATGGAACCTGCTTAAGCCACACATCAGGAGGCCAGAGCCTCTATATTTTGCTTGTGGGCACAGTGAAAAGTTTATTTTATGCATGTTAAATCCTGCATACAAAACAACATATCTGGTCATATGGCTCTCATTAGCTCCCAATTTTGCACATTTTATCCAACTTGTCATGAGAACTGGGCTTATGCTTCAACTGTAGTCCTTCAAACAACCCTTCAC**CG**TTCTTGTCAGAGGACCCCTTCCTCCTGTGTTATTTACCAGACTTGCCAACACCCCTCCCCCACAAAAAAAGCCTGACCAGCTTTACCCCTATTTCAAGGAGTGTTCACTTCTTTTCATTAATCTGCTGTTATATTTGTAACAATTTAGAAATTATAGGAGTTGAAGTTCTGGTAAAAAAGAATGATGCATGAGGGTTTTTGTTGTTGTTGTTGTTGTGTGTGTGTGTGTGTGTGTGTGTGTTTGGTTAGTTAGAATGAGATTCTAAAGACCTGGGAAGGATACTTATGAAGTTCATTTAAAGAGAATTACTATTTCCAAAGTTCTTGATTTATGAAGCAGGCATTCAATTTCAAGATGTAAAATAATAGTGTAAGTTGGTTGGGGT**CG**GGGGAGACCATCATCATATGATTATACAGATTTTCCCAATTTTTTTCCTCAATGAAATAGCTTATTTTAAAAATATAAAATTAGAATCCCTATAAAGAACACTACCAATTTTCTTTCCTGAAAGAAACAATAAAATACTTTGTAAATAGAAGCAACTTCATGAAGTAACAGATGTTTTATGTAAAGACATAAAACTGAACCTGAATATAA**CG**AGA**CG**TGTGTG**CG**AGTAGTAAAAGTTGAAAGTTGGACACACTCATTGAGACATATCTATTTGATTCCAATGTTTTTCTAAAAGGTAGAGTAATCCTAGCCAGAGGTTTCACTGGCTCAGTGCATCACCCAGTAGTGTCTCAGAAGCCAGGAAGGGCTTTCCATTAGATAATGAATTATGAAATGTCTCACACTGGAAAAACCAGTCATC**CG**CTGATGTCATGCTGATTCCAACCAATCCCAAACAAAGCCCCAGCCCTCCTCTGTTTCAGTGGTACCAATGTGTGGTGTACAAATAAGTAGTACAGTATAAAACTTCACAGTGCCAATACCATGAAGAGGAGCTCAGACAGCTCTTACCACATGATACAAGAGC**CG**GCTGGTGGAAGAGTGGGGACCAGAAAGGTAATGCTTTTTAACTCTTACTTCTGAGCTCTTTACACATTCAAAGATAGGAAAGCTAGGAGGAATTTTACAACTAATTGGCATTTCCAATGTGCATTGTGATGTGTACCTTTTTATATTATTCAGGCAGGTTAATACAGCTTTTAATAGTCCTAGAGCATGCAAATAGATTATATGTTTATACAAGCCACTCAGCACATATATACAAGTACATATGCCAAAGAGAAAGCTATTTTTAAGAGTTACATT**CG**CAAACAGTAAATTCAGGGAACACACACATACTCAGATGCAGAGAGAATCCAAATATTGATAAGTTGCACTTATCTAAATGCTGCTATTAGGACTCCTGAGTTGTTTAGAGCCATTAAACTTTTGGTTGTATTTCAGACTTTCTTGTAAAACTTAATTGAACTGCAAAACATTTTGGGTACTGTATATGTGACTCCAAATAGGTGGATGATGTTAAGTATTATAGCACAAAGATTTTTTATAAAACCATTGTAACACAAATGTCCCCTGCCTCCCCCATTCTCTTTCACCATCCC**CG**TAAAAAATATGAGGCTTTTTAGGCAATGTTGACAAAGTTTTAACAATAATGGTGGAGTAATTGATGTTTCTGGAGCTGAAACCCCAAAGGTGTTAGGTTACTTGTAACAGAAAAAGTCTTACAAATAGTTGTTTTGGAAAAGGGGACAGTATATATAATTCAGAAAGCATTGTTAACCTGTGCAAACTGTAATTATAACTTAGTGTCACAATTTTCTTGCCCTCTTCCCCTTGCACTCAGATCTTATCTTGTAGTAATGATATTTATTAACTCTTTCCACCTAAACTGCATTGCTTGACTGTAATGCTATGAACACACTAGGTGTCAGATATAAGCTGAGTGTATCTTCAGAAACCAAGAGGGCTTATGTGTGGGAAAGAAAC**CG**AGAGGGAAGGAA**CG**CTTTAACAGATGGACCCCTTAAAGATTCTTCTGCAAGATAAAAGCAATAAGACAGAAAATGAAAAAGAGGGGAGGGGGAAGAATTTTTTTTAAGCCTTAGAAAGGCATTGTTAAAAAATTCACATTTTTCTTTTTCTGTGCACACTAAAATCCATGATGATTTCATCTGCACTGTTCCTTTGAGGGAAAAAGAAGCAGTCAAGGAGGCTGTCTATGAATGCACTGGT**CG**GGACAGGCTTGGGGCAAGCTGAAAAAACTACCACATGACAGAGAAAAATAATTTGCCAATATATTTTAGAGAGTCTTTTCCCATAGGACCAGTTATTCAAGTCATA**CG**AGTGCACTCTTTTTATAAAAGGATGTGGGAAAGGCCAAGAGAATTTTGCATTTTATCTGTGAAGTC**CG**G**CG**AGTGGTGGTAGGCTGTAATGTGTGAGAGTGAGTGGGTC  CC**CG**GCAGAAGGGGGCAGCTGAA**CGCG**A**CG**GGGAGAAAG**CG**CTTCTGGAACTTGGGCTTGTGACAGGCTGCCTGCCCTCTGGTCCTTCAGTGCCCTGCTGCATTTCACAGTTGGGAAGAGTGGAGTGTATTATATGACCCCAAACAAAAGTTCCATTG**CG**CTCTCCTCAGATTGCCTGCCAGTGTTGATGACCTGAACATTTAAATATGAATGAATTGGGGGAAAGGACCATCTCCCCTGGATCCCATCAGGCCAGAACAATCCTCTGTTACCCCTGAGTCCCTCTTCCCTGACCTCCACTACCTGTCCACTGGACCTCCCTGCACCCTCTGCCCCACCA**CG**TGGCCAGGTGGG**CG**TTCTACCACCTAGGGCTGTGGCTTGGCTGGGTGGAGGGG**CG**GTGGGAACACTTTTTCAATCAATTCATCCCTATTGATTGAGACACTGTGTTTGTTTGGGGTTTCTTTTCTCCCCTCCAAAAAAGGAAGAAGGTGAAACCAGGAGACTGGGCAGAGAAAGAAAAAAAAATAGTGAACAAAATTAGGATAATTTATTTTAGAAGAGAAAGTAGAACCCC**CG**AAAATGGTGATATTTGAAGAGAGGTGTCTGTGAGGAAGCTAAGAGCAGAAGGAGAGCAGCCTGTCAGAAAA**CG**GGCTGTCCCTCCCTCCCTAATCACAGCCCTACTCACAGCAAACTCCCTCCCTCTCCATTCACTCACTTACTTAGGGCCAATCCTTTCTCTCTCTCTCTCTCTCTCTCTCTCTCTCTCTCTCTTCCTTTCCCTCTCTCCCTCTCCCCCTCCCTTCCCTCCCTCTCTCCCTCTCTCTCCCCCTTCTTTCCCTCTCTCTTCTCTCCCCCTCTCTCCTCTCTCTCTGTCTCTGTCTCCCTCCCATCCTCTCTCTCTGTCTCTGTCTGTCTCCC**CG**CCACCCTGTCTCTCCCTCCCTCCCTGTCTCCCTCCCTCCCTCCCTCCTTCCTTCTCTCTTACT**CG**GAGACAGTCAGAACTCTCCTCCCTGACAGCCACAAACCTACAGCACTGACTGCATTCAGAGAGGAACCTGCAAACAAAACTTCACAGAAAACTTTTTGTTCTTGTTCCAGAGAATTTGCTGAAGAGGAGAAGGAAAAAAAAAACACCAAAAAAAAAAATAAAAAAATCCACACACACAAAAAAACCTG**CGCG**TGAGGGGGGAGGAAAAGCAGGGCCTTTTAAAAAGGCAATCACAACAACTTTTGCTGCCAGGATGCCCTTGCTTTGGCTGAGAGGATTTCTGTTGGCAAGTTGCTGGATTATAGTGAGGAGTTCCCCCACCCCAGGATC**CG**AGGGGCACAG**CG**CGGCCCC**CG**ACTGTC**CG**TCCTGTG**CG**CTGGC**CG**CCCTCCCAAAGGATGTACCCAACTCTCAGCCAGAGATGGTGGAGGC**CG**TCAAGAAGCACATTTTAAACATGCTGCACTTGAAGAAGAGACC**CG**ATGTCACCCAGC**CG**GTACCCAAGG**CG**G**CG**CTTCTGAA**CGCG**ATCAGAAAGCTTCATGTGGGCAAAGT**CG**GGGAGAA**CG**GGTATGTGGAGATAGAGGATGACATTGGAAGGAGGGCAGAAATGAATGAACTTATGGAGCAGACCT**CG**GAGATCATCA**CG**TTTGC**CG**AGTCAGGTTGGTGCTGGCATTGGCAGGGGGTGGGGAGGGGTGGGGGGTGGGAGGGTAAAATATATTTCTTTGACAGTCCCAGGAGGAACTTCTTTTCCCTTCAGCTGGAAACTGCCTGGGAAGGTTATTAGTTATTAGGTGATGGTAG**CG**GACTAGC**CG**A**CG**GAGGGCAGGCAGGGGAGGGGGAGAGGACTTTACAGAAAAGGAATTCT**CG**GT**CG**AGCTCTGCCTGGAGATGACTGGCTTACACTTACTAAACCCAG**CG**GGTCACACAGAGAGGAAGCT**CG**GGGCCAATGTTGAGCTGGAAGGCAGACTGTGAGGGGCTGCCTTGCCCTGCCTGTGAAACCAGATCTGAGCAGC**CG**GAGGAAAGC**CGCG**GCATTTT**CG**GGTGCTAGGGGAGCAGAGGAGGCTTC**CG**GACCCCATCCAAGTTTTTATTGAGGGTAGAGGGGTGAATGTACCAGGATTGGAGTGGAATGGCACAGATGAAGTCACTCTCTTAAAACAAACCTTCCCCTTTAAAAGTCCAATCTGGGGCCACATTGGAGAAGCAGGGCATATTTATGAGTGACAGTCATTTTTACCTTTAGAAAATGTCTATAAGTGCACAGGCACCACATTCAAGACAGGGAAGAGCTACTTTGGGGGACAGTTGTCATTGAACCAGCAGTTACTTTTGGGACACTGACTTTTGCTCTCTGAAAGAAAAAAAAATAAATAAAACAACCAGTTTTGTTCTTTCTAAAGTTACTAAGAGCTCTCTGCCAAGGAA |

The underlined sequence is where CpG islands cluster, located in the first exon region. The three sequences in square columns are the positions corresponding to the three primers, including 26 loci in total.
