# Supplementary material for: DNA hypomethylation of INHBA promotes tumor progression and predicts prognosis and immune status of gastric cancer
Source: Hereditas. 2024 Nov 14;161:45. doi: 10.1186/s41065-024-00347-7 (PMC11562481; doi:10.1186/s41065-024-00347-7)
Supplement: Supplementary file 6 — Supplementary Material 6 [file 41065_2024_347_MOESM6_ESM.docx]

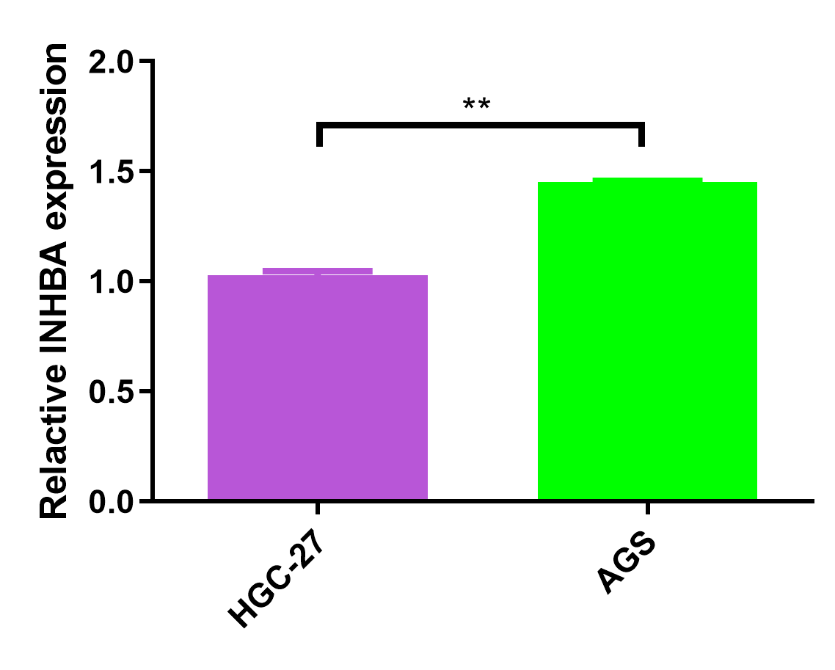


**Supplementary Figure 1. The relative INHBA mRNA expressions of HGC27 and AGS**


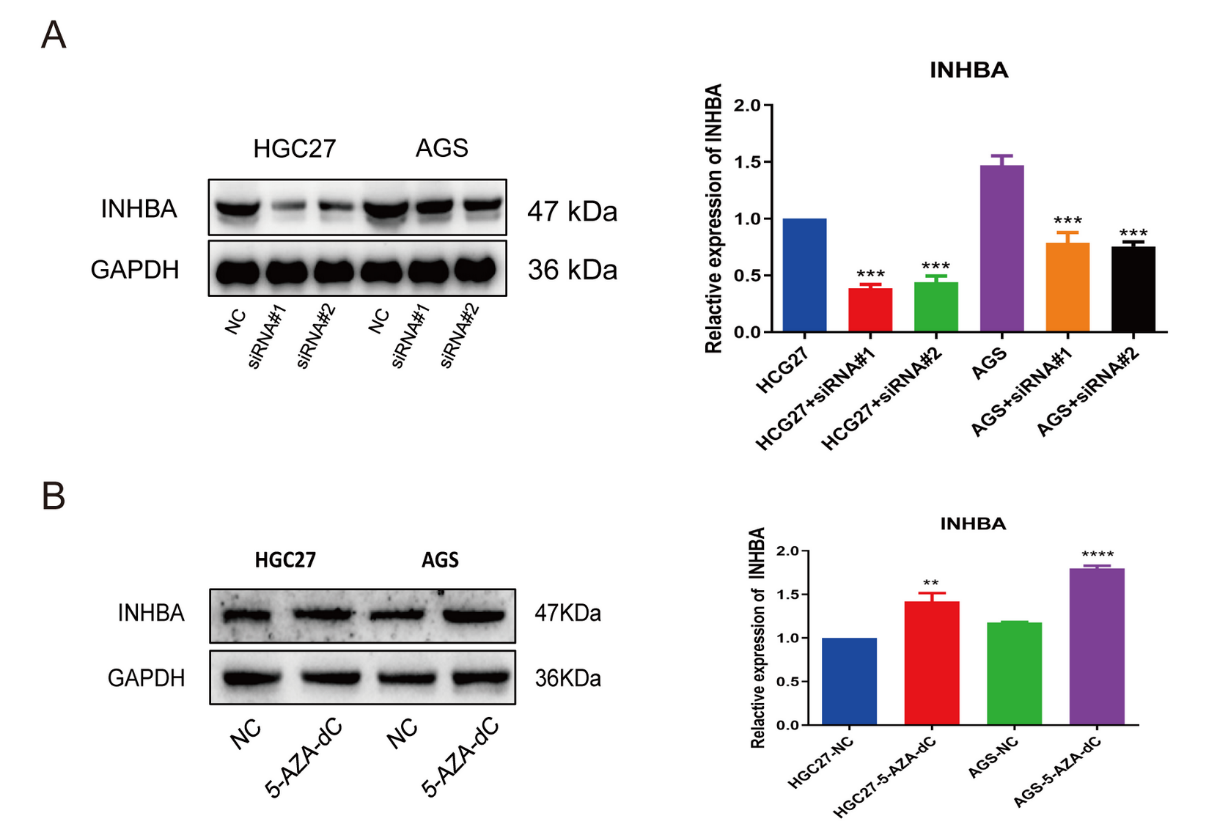


**Supplementary Figure 2. The expression of INHBA protein.** A. The efficiency of INHBA knockdown was tested. B. The expression of INHBA increased after 5-AZA-dC treatment. (** p＜0.01, *** p＜0.001, **** p＜0.0001; *t*-test)


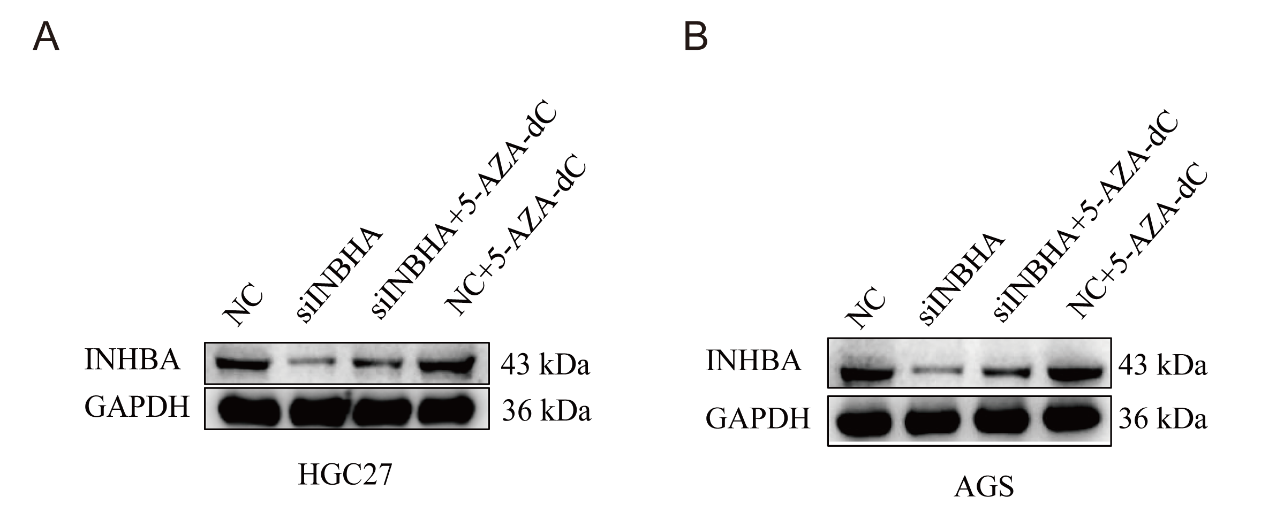


**Supplementary Figure 3.** **The expression of INHBA protein in the rescue experiments.** A. HCG27. B.AGS.
