# Supplementary material for: DNA hypomethylation of INHBA promotes tumor progression and predicts prognosis and immune status of gastric cancer
Source: Hereditas. 2024 Nov 14;161:45. doi: 10.1186/s41065-024-00347-7 (PMC11562481; doi:10.1186/s41065-024-00347-7)
Supplement: Supplementary file 7 — Supplementary Material 7 [file 41065_2024_347_MOESM7_ESM.docx]

**Cell culture**

Human gastric cancer cell lines HGC-27 (CSTR:19375.09.3101HUMTCHu22) and AGS (CSTR: 19375.09.3101HUMTCHu232) were obtained from Shanghai Institutes of Biological Sciences (Shanghai, China). All cells were routinely cultured in the Roswell Park Memorial Institute (PRMI) 1640, supplemented with 10% fetal bovine serum (FBS, BI), penicillin (100units/ml) and streptomycin (100g/ml), and cultured in a thermostatic incubator (Thermo Scientific, USA) with a humidified environment of 5% CO_2_ and 37 ℃.

**RNA extraction and RT-qPCR**

Total RNA was isolated using TaKaRa MiniBEST Universal RNA Extraction Kit and cDNA was synthesized with TaKaRa Primescript RT reagent kit with gDNA Eraser (perfect Real time) according to the manufacturer’s protocol. Bio-Rad QX100 Droplet Digital PCR system (USA) was used to measure expression RNA by SYBR Green (Roche). The method for calculating the relative RNA amount was described in our previously study. All premiers were obtained from Tsingke Biological Technology (Beijing, China) and presented in Supplementary Table 2.

**Western blotting**

Total proteins were extracted from cells using pre-cooled RIPA buffer (manufactured by Beyotime in Shanghai, China) that contained protease and phosphatase inhibitors (supplied by Thermo Scientific in the USA). The quantification of proteins was carried out using the Bicinchoninic Acid protein assay kit (supplied by Thermo Scientific in the USA). The protein samples were separated equally using SDS-PAGE (manufactured by SEVEN BIOTECH in Beijing, China) and then they were transferred to a 0.45 μm PVDF membrane (supplied by Millipore in the USA). Following a 1-hour blocking with 5% skim milk in TBST buffer, the membrane was exposed to the relevant primary antibody at 4℃ overnight. Subsequently, the membrane was rinsed with TBST buffer three times, and then incubated with the enzyme-labeled secondary antibody at room temperature for 1 hour. Western blotting was carried out using an imaging system (made by Bio-Rad in the USA) and an enhanced chemiluminescence detection kit (supplied by Servicebio in Wuhan, China). GAPDH was used as the loading control. The relative level of protein expression was calculated by Image J. All the antibodies used in the study were listed in Supplementary Table 3.

**RNA interference**

Shangya Biotechnology (Hangzhou, China) synthesized small interfering RNAs (siRNAs) targeting INHBA, as well as negative control RNAs (siNC). The manufacturer's protocol was followed for transient transfection using the jetPRIME Polyplus Kit (France) All the siRNA sequences were summarized in Supplementary Table 4.

**Cell proliferation assay, colony formation and EdU incorporation assay.**

The assessment of cell proliferation ability was conducted using the Cell Counting Kit-8, following the manufacturer's instructions. For the colony formation assay, between 1.0× 10^3^ cells (for the rescue experiment) and 2.0× 10^3^ cells (for the cell functional experiment) were seeded in 6-well cell culture plates, with three replicates. After allowing the cells to incubate for two weeks, the plates were fixed with paraformaldehyde and stained with 1% crystal violet for a duration of 10 minutes. In addition, the proliferation ability of gastric cancer cells was also evaluated by means of an Edu assay, which involved the use of a 5-ethynyl-20-deoxyuridine (EdU) assay kit (Ribobio, Guangzhou, China). The assay was conducted in compliance with the manufacturer's protocol and the results were observed via a fluorescence microscope.

**Migration and invasion assays**

During migration and invasion assays, a 24-well plate was utilized alongside a transwell filter insert (Corning, NY, USA) comprising a pore size of 8 μm. In the context of the invasion experiment, a diluted matrix was added to the transwell filter insert beforehand. Approximately 1 × 10^5^ gastric cancer cells, suspended in serum-free medium, were placed in the upper cavity of the insert and a medium containing 10% fetal bovine serum was added to the lower cavity. The culture plates were incubated at 37 °C for 24 hours (for the rescue experiment), and 48 hours (for the cell functional experiment) before fixing and staining the submembranous cells with crystal violet. Finally, cell counts within five randomly selected regions were performed under a microscope.
